# A Novel Phosphoregulatory Switch Controls the Activity and Function of the Major Catalytic Subunit of Protein Kinase A in *Aspergillus fumigatus*

**DOI:** 10.1128/mBio.02319-16

**Published:** 2017-02-07

**Authors:** E. Keats Shwab, Praveen R. Juvvadi, Greg Waitt, Erik J. Soderblom, M. Arthur Moseley, Nathan I. Nicely, Yohannes G. Asfaw, William J. Steinbach

**Affiliations:** a Division of Pediatric Infectious Diseases, Department of Pediatrics, Duke University Medical Center, Durham, North Carolina, USA; b Duke Proteomics and Metabolomics Core Facility, Center for Genomic and Computational Biology, Duke University, Durham, North Carolina, USA; c Duke Human Vaccine Institute, Duke University School of Medicine, Durham, North Carolina, USA; d Department of Laboratory Animal Resources, Duke University Medical Center, Durham, North Carolina, USA; e Department of Molecular Genetics and Microbiology, Duke University Medical Center, Durham, North Carolina, USA; Washington University School of Medicine

## Abstract

Invasive aspergillosis (IA), caused by the filamentous fungal pathogen *Aspergillus fumigatus*, is a major cause of death among immunocompromised patients. The cyclic AMP/protein kinase A (PKA) signaling pathway is essential for hyphal growth and virulence of *A. fumigatus*, but the mechanism of regulation of PKA remains largely unknown. Here, we discovered a novel mechanism for the regulation of PKA activity in *A. fumigatus* via phosphorylation of key residues within the major catalytic subunit, PkaC1. Phosphopeptide enrichment and tandem mass spectrometry revealed the phosphorylation of PkaC1 at four sites (S175, T331, T333, and T337) with implications for important and diverse roles in the regulation of *A. fumigatus* PKA. While the phosphorylation at one of the residues (T333) is conserved in other species, the identification of three other residues represents previously unknown PKA phosphoregulation in *A. fumigatus*. Site-directed mutagenesis of the phosphorylated residues to mimic or prevent phosphorylation revealed dramatic effects on kinase activity, growth, conidiation, cell wall stress response, and virulence in both invertebrate and murine infection models. Three-dimensional structural modeling of *A. fumigatus* PkaC1 substantiated the positive or negative regulatory roles for specific residues. Suppression of PKA activity also led to downregulation of PkaC1 protein levels in an apparent novel negative-feedback mechanism. Taken together, we propose a model in which PkaC1 phosphorylation both positively and negatively modulates its activity. These findings pave the way for future discovery of fungus-specific aspects of this key signaling network.

## IMPORTANCE

Our understanding of signal transduction networks in pathogenic fungi is limited, despite the increase in invasive fungal infections and rising mortality rates in the immunosuppressed patient population. Because PKA is known to be essential for hyphal growth and virulence of *A. fumigatus*, we sought to identify fungus-specific regulatory mechanisms governing PKA activity. In this study, we identify, for the first time, a novel mechanism for the regulation of PKA signaling in which differential phosphorylation of the PkaC1 catalytic subunit can lead to either positive or negative regulation of activity. Furthermore, we show that inactivation of PKA signaling leads to downregulation of catalytic subunit protein levels in a negative-feedback mechanism distinct from expression patterns previously reported in the yeasts. Our findings represent a divergence in the regulation of PKA signaling in *A. fumigatus*, which could potentially be exploited as a target and also open the avenue for discovery of fungus-specific downstream effectors of PKA.

## INTRODUCTION

Cyclic AMP (cAMP) signaling, mediated by protein kinase A (PKA) in eukaryotes, represents a vital sensory pathway that is conserved across all domains of life (see references 1–3 for reviews). The filamentous fungus *Aspergillus fumigatus* is one of the leading infectious killers in immunocompromised patients (4–6). Deletion of either the *A. fumigatus* primary PKA catalytic subunit (PkaC1) or the regulatory subunit (PkaR) has been shown to inhibit growth and conidiation of the fungus and leads to attenuated virulence in a murine model of invasive aspergillosis (IA) (7–9). To date, no studies have defined the specific mechanisms by which PKA activity is regulated in this important pathogen.

In addition to activation through cAMP binding of the regulatory subunit, in a variety of species activation of PKA has been found to also require phosphorylation of the catalytic subunit at specific amino acid residues. Of two phosphorylation sites identified in the natively expressed mammalian PKA catalytic subunit (10, 11), only T197 is conserved in fungi. T197 represents a highly conserved phosphorylation site common to AGC family kinases, sometimes referred to as the T-loop residue (12). This residue is located within the activation segment of the enzyme, and its phosphorylation is required for PKA function (13–15). In the yeasts *Saccharomyces cerevisiae* and *Schizosaccharomyces pombe*, phosphorylation of the T-loop residue has also been identified as essential for effective PKA signaling, impacting regulatory subunit binding and catalytic efficiency (16–19). The presence of other phosphorylated residues on the *S. cerevisiae* catalytic subunit besides the T-loop residue has been deduced experimentally, but neither have these specific sites been identified nor are their functions deciphered (16). In *S. pombe*, Pka1 has been found to be hyperphosphorylated at unidentified sites in the absence of cAMP (18, 20). This hyperphosphorylation is induced by glucose starvation associated with reduced cAMP levels and during osmotic stress, a condition under which PKA has been shown to be necessary for *S. pombe* growth, suggesting that hyperphosphorylation may be a means of maintaining some degree of PKA activity during particular stresses when cAMP levels are low (20). Beyond these few insights, phosphoregulation of PKA in fungi remains largely mysterious.

In the current study, we used liquid chromatography-tandem mass spectrometry (LC-MS/MS) to identify specific phosphorylated residues in the PkaC1 protein of *A. fumigatus*, including three sites not previously identified as phosphorylated in PKA isoforms of mammals or any other fungal species. We demonstrate the key importance of phosphorylation at these sites in regulating growth, conidiation, stress tolerance, and virulence and propose a novel model of PKA regulation in this human pathogen.

## RESULTS

### *A. fumigatus* PkaC1 is phosphorylated at novel sites and localizes in the cytoplasm under normal and cell wall stress-inducing growth conditions.

**FIG 1:**
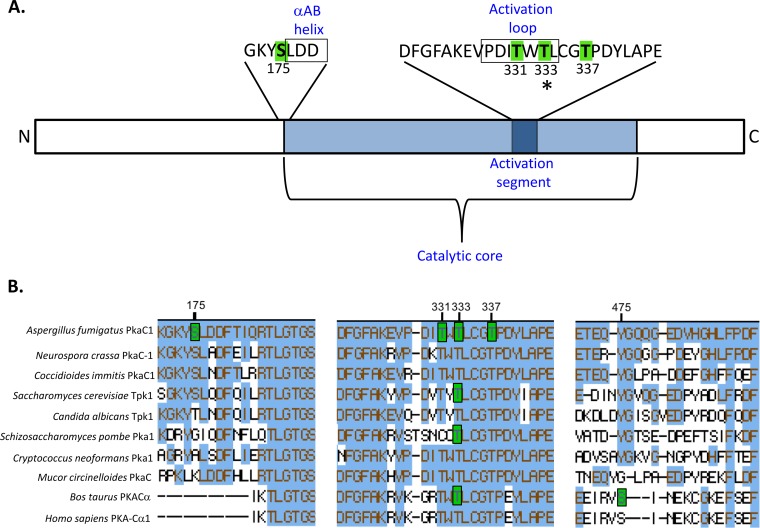
Multiple sites of phosphorylation identified in PkaC1 by mass spectrometry. (A) Diagram showing locations of phosphorylated sites within the PkaC1 protein. Residues highlighted in green were identified by LC-MS/MS as phosphorylated. Serine 175 is located immediately outside the conserved catalytic core and adjacent to a region with homology to the αAB helix (boxed) identified in the *S. cerevisiae* Tpk1 crystal structure. Threonine residues at positions 331, 333, and 337 are located in the conserved activation segment of the catalytic core containing residues essential for catalytic activity of PKA. T331 and T333 are located within a subdomain of this segment known as the activation loop (boxed). The asterisk at T333 indicates the conservation in phosphorylation of this residue in other species. (B) Clustal W alignment of phosphorylated regions of PKA catalytic subunits of selected fungal and mammalian species. Amino acids highlighted in blue indicate their conservation in relation to *A. fumigatus* PkaC1. Amino acids highlighted in green indicate residues that have been experimentally determined to undergo phosphorylation in the associated organism. Numbering of the residues showing the respective phosphorylation is based on the *A. fumigatus* protein. Of the two phosphorylated sites identified in mammalian PKA, only one site corresponding to residue T333 is conserved in *A. fumigatus*. S175 is conserved among some fungal species but not in mammalian enzymes. T333 and T337 are conserved in all species analyzed, while T331 is conserved in all except *S. pombe*.

In order to assess the phosphorylation state of PkaC1 during growth under optimal and cell wall stress conditions, an *A. fumigatus* strain expressing PkaC1-green fluorescent protein (GFP) fusion protein was engineered and cultured in the presence or absence of caspofungin (CSP; 1 µg/ml). The PkaC1-GFP fusion protein was purified by GFP-Trap affinity purification and subjected to LC-MS/MS analysis. PkaC1 was phosphorylated at four residues (Table 1; Fig. 1A; see also Fig. S1 in the supplemental material), including a single serine residue at position 175 (S175) and three threonine residues at positions 331, 333, and 337 (T331, T333, and T337, respectively). Phosphorylation at these sites was independent of CSP treatment (data not shown). Amino acid sequence alignments revealed that the three threonine residues (T331, T333, and T337) are conserved in other PKA catalytic subunit isoforms, including those of *S. cerevisiae* and humans, while S175 is found only in some related species of ascomycetes (Fig. 1B). S175 is located at the N-terminal end of the conserved catalytic core region, immediately adjacent to the short αAB helix, while T331, T333, and T337 are located within the activation segment of the enzyme, with T331 and T333 positioned within the activation loop (Fig. 1A). T333 represents the T-loop residue of *A. fumigatus*, homologous to T197 in mammals and T241 in *S. cerevisiae*, and thus, phosphorylation at this site is consistent with findings from these and other species. However, phosphorylation at the other three residues (S175, T331, and T337) has not been reported for any PKA catalytic subunit isoforms in other species and thus may represent a novel mechanism for the regulation of PKA activity.

10.1128/mBio.02319-16.1 FIG S1  Spectra indicating phosphorylation at specific PkaC1 residues. Tandem mass spectra of GKY[pS]LDDFTIQR and EVPDI[pT]W[pT]LCG[pT]PDYLAP from PkaC1 reveal four unique phosphorylated residues including one serine (S175) and three threonines (T331, T333, and T337) in close proximity. The presence of each identified C-terminal (y) and N-terminal (b) product ion is indicated within the peptide sequence. Peaks indicating phosphorylation are represented in yellow. Download FIG S1, PDF file, 0.3 MB. Copyright © 2017 Shwab et al. 2017 Shwab et al. This content is distributed under the terms of the Creative Commons Attribution 4.0 International license.

**TABLE 1:** PkaC1 phosphorylation sites identified by LC-MS/MS analysis

Peptide sequence^a^	Phosphorylated residue	*m/z*	Charge	Mass error (ppm)	Mascot ion score^b^	Ascore localization probability^c^
GKY[pS]LDDFTIQR	S175	761.8521	2	2.11	49.5	99.99
EVPDI[pT]WTLCGTPDYLAP	T331	919.7646	3	2.29	52.8	95.22
EVPDITW[pT]LCGTPDYLAP	T333	1,379.1445	2	1.1	73.9	95.74
EVPDITWTLCG[pT]PDYLAP	T337	919.765	3	2.75	57.1	99.36

a “[pS]” or “[pT]” indicates a phosphorylated residue.

b Mascot identity score of >41 indicates identity or extensive homology (*P* < 0.05).

c Probability of phosphorylated residue localization based on Ascore algorithm. Peptides contained uniquely identified phosphorylation residues or combinations of phosphorylation residues. Due to the proximity of multiple phosphorylatable residues within these peptides, mass spectra from each identification were submitted to an independent algorithm (Ascore) which assigns confidences to the localization of each phosphorylation.

Localization of PKA catalytic subunits was found to alternate between the cytosol and nucleus depending on the presence of environmental stressors in the yeasts *S. cerevisiae*, *S. pombe*, and *Candida albicans* (18, 21–24). In order to determine whether similar localization patterns occur in *A. fumigatus*, the PkaC1-GFP strain was cultured in the presence of 0 to 1 µg/ml CSP. Under all growth conditions tested, localization of PkaC1-GFP fusion protein appeared to be primarily cytosolic (Fig. S2), and the presence of CSP did not affect localization.

10.1128/mBio.02319-16.2 FIG S2  Localization of PkaC1 during caspofungin exposure. Conidia (10^4^) of the strain expressing GFP-labeled PkaC1 (PkaC1-GFP) were cultured on coverslips immersed in GMM broth with or without the addition of the indicated concentrations of caspofungin (CSP) and incubated for 20 h at 37°C. Hyphae were visualized using an Axioskop 2 Plus microscope (Zeiss) equipped with AxioVision 4.6 imaging software. Differential interference contrast (DIC) images are shown in grayscale, while GFP fluorescence is shown in green. In all samples, PkaC1 appears to be localized in the cytosol. Download FIG S2, PDF file, 0.2 MB. Copyright © 2017 Shwab et al. 2017 Shwab et al. This content is distributed under the terms of the Creative Commons Attribution 4.0 International license.

### Structural modeling of *A. fumigatus* PkaC1 signifies key regulatory roles for phosphorylation in PkaC1 function and activation.

**FIG 2:**
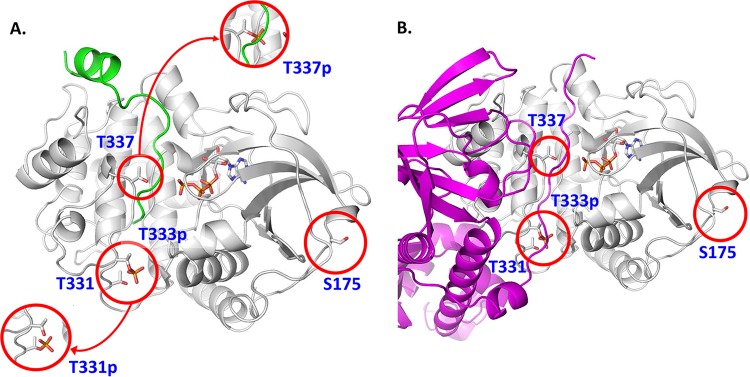
*A. fumigatus* PKA structural model showing the position of phosphorylated residues that may disrupt PKA activity and function. (A) The *A. fumigatus* PKA sequence was threaded onto the *S. cerevisiae* source model (39), with which it shares 48% sequence identity. The backbone of the structure is shown in white, with the bound substrate peptide shown in green and the ATP cofactor depicted in atom colors. Phosphorylation of T331 (T331p, lower inset) would likely preclude phosphorylation of the nearby typically constitutively phosphorylated T333 (T333p) as rendered and would cause structural disruptions to the distal end of the substrate binding site. Phosphorylation of T337 (T337p, upper inset) would cause significant steric hindrance to bound peptides and proteins as well as strong charge repulsion with the triphosphate moiety of the ATP cofactor. (B) The *A. fumigatus* regulatory protein sequence was threaded onto a *Mus musculus* source model (40) and is shown in magenta. Part of the N terminus of the regulatory subunit, the “inhibitor sequence,” mimics substrate in the PKA binding site, and as such, transient phosphorylation of T331 and T337 would cause similar structural disruptions, thus breaking important protein-protein contacts in the holoenzyme.

In order to assess the functional impact of phosphorylation at the four residues identified in PkaC1, a three-dimensional structural model of the enzyme was generated by threading onto the known three-dimensional structure of substrate-bound *S. cerevisiae* PKA catalytic subunit TPK1, which has the highest homology to *A. fumigatus* PkaC1. As the *S. cerevisiae* and *A. fumigatus* proteins are highly conserved within the catalytic core region, we expected this approach to provide a close approximation of the true structure. Figure 2A depicts the predicted *A. fumigatus* PkaC1 structure and highlights the positions of the phosphorylated residues in substrate-bound PkaC1. As only the T-loop residue is phosphorylated in the *S. cerevisiae* TPK1, T333 is shown in the phosphorylated state in Fig. 2A, while the other sites are shown in the dephosphorylated state, with insets depicting phosphorylated versions of T331 and T337. T333 is located at the base of the substrate binding cleft, where its phosphorylation may facilitate interactions with a number of proximal residues, stabilizing the cleft and anchoring the substrate. T331 is adjacent to T333, so that simultaneous phosphorylation at the two residues would be unstable, meaning that T331 phosphorylation would likely preclude phosphorylation at the T-loop residue, destabilizing substrate binding and potentially interfering with enzyme function. T337 is predicted to be located near the site of phosphate transfer from bound ATP to substrate, so that interaction of this residue with key catalytic residues is likely, and phosphorylation here would likely be disruptive to the catalytic process as well as to substrate binding. Therefore, phosphorylation at both T331 and T337 may represent mechanisms for negative regulation of PKA activity. S175 is located on the external surface of the folded protein, distal to the active site. As such, phosphorylation at this residue would not be expected to have a direct impact on catalytic function of the enzyme, though it is conceivable that this could have an influence on other potentially important protein-protein interactions. The effects on substrate binding predicted for T331, T333, and T337 would also potentially impact binding to the PKA regulatory subunit, which is predicted to mimic the structure of the substrate and to bind to the catalytic subunit in approximately the same region (Fig. 2B).

### Mutation of *A. fumigatus* PkaC1 phosphorylated residues in the activation segment drastically alters PKA activity.

**FIG 3:**
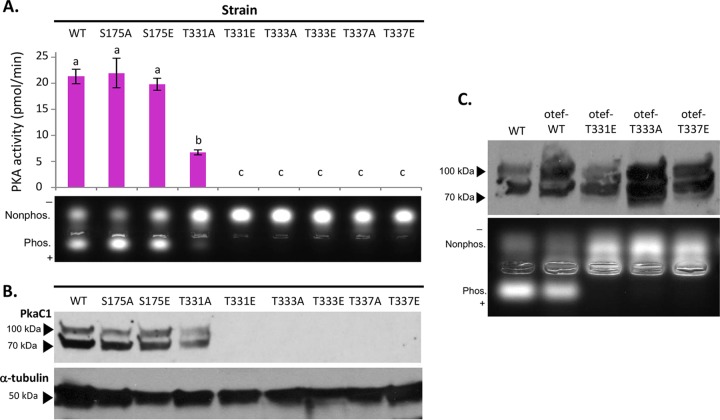
Impact of PkaC1 phosphomutations on protein kinase activity. (A) Protein normalized crude cellular extracts for each mutant strain were added in duplicate to reaction mixtures containing fluorescent dye-coupled PepTag peptide (Promega) as a PKA-specific test substrate and incubated for 30 min at room temperature. Activity was assessed qualitatively based on migration of negatively charged, phosphorylated peptide toward the anode of an agarose gel (lower panel) and quantified by comparing the fluorescence of a phosphorylated substrate for each sample to a standard curve using a plate reader, and the average values for each mutant were plotted (upper panel). Activity was defined as picomoles of substrate phosphorylated per minute. Error bars represent standard errors. Activities were analyzed via Student’s *t* test, and statistically significant differences (*P* < 0.05) between samples are indicated by different letters above columns. S175 mutants showed activity similar to that of the wild-type (WT) strain, while the T331A strain had significantly reduced activity and the remaining mutants had no detectable activity. (B) Proteins from the same normalized crude extracts used for the kinase assay were separated via SDS-PAGE (4 to 20%), hybridized to a PVDF membrane, and then probed with anti-GFP primary antibodies to detect PkaC1 isoforms and anti-α-tubulin antibodies as an internal control in Western blot assays. Full-length PkaC1 protein of approximately 80 kDa was detected for WT, S175A, S175E, and T331A samples, but no protein was detected in any of the other samples. The higher-molecular-mass band likely represents an alternate phosphorylation state. (C) Crude extracts from WT and strains overexpressing PkaC1 with native (WT) sequence or the T331E, T333A, and T337E mutations via the constitutive *otef* promoter were probed in a Western blot assay as described above (upper panel) and also tested for PKA activity as described above (lower panel). While full-length protein was detected in all samples, kinase activity was detected only in samples expressing WT protein.

In order to obtain a clear mechanistic understanding of the *A. fumigatus* PkaC1 structure versus function relationship and associate our molecular modeling data with enzyme activity, we next introduced both phosphomimicking (glutamate substitution) and nonphosphorylatable mutations (alanine substitution) at the four phosphorylated residues in PkaC1 (S175, T331, T333, and T337) and expressed the respective constructs under the control of the *pkaC1* native promoter in *A. fumigatus*. In all strains used in the experiments described here (see Table S1), PkaC1 isoforms expressed are C terminally labeled with GFP in order to facilitate detection and purification. The PkaC1-GFP wild-type (WT) strain served as a positive control and showed no phenotypic differences from the *akuB*^*KU80*^ parent strain with regard to radial growth, conidiation, antifungal susceptibility, or PKA kinase activity (data not shown). PKA activity was assessed based on the ability of PkaC1 to phosphorylate a fluorescently labeled substrate peptide (PepTag A1 peptide LRRASLG; Promega) with high specificity. As a measure of activity, migration of the negatively charged phosphorylated peptide was visualized on an agarose gel and quantified spectrophotometrically (Fig. 3A). As shown in Fig. 3A (lower panel), the phosphorylated peptide (showing downward migration) was abundant in samples treated with extracts from the WT, S175A, and S175E strains and was visible to a lesser extent in the T331A strain. No phosphorylated peptide was detected in the samples treated with extracts from the T331E, T333A, T333E, T337A, or T337E mutant-expressing strains, as in the Δ*pkaC1* negative-control strain (data not shown), indicating a pronounced reduction in PKA activity due to these mutations. Quantification of the excised phosphorylated peptide bands revealed no statistically significant difference between the S175A and S175E substitution strains compared to the WT control (Fig. 3A, upper panel). Extracts from all other strains tested were found to have produced significantly less phosphorylated peptide than the WT control (*P* < 0.05). Phosphorylation by the S175A, S175E, and T331A strains was significantly higher than that in the Δ*pkaC1* negative control, while no significant difference could be measured between the PKA activity of the negative control and the remaining strains, none of which produced a detectable level of phosphorylated substrate peptide.

10.1128/mBio.02319-16.3 TABLE S1  *A. fumigatus* strains used in this study. Listing and genetic description of *A. fumigatus* strains used in the described experimentation. Download TABLE S1, DOCX file, 0.02 MB. Copyright © 2017 Shwab et al. 2017 Shwab et al. This content is distributed under the terms of the Creative Commons Attribution 4.0 International license.

Next, in order to ascertain whether the observed reduction in PKA activity in the mutant strains was due to the nonfunctionality or degradation of the mutant proteins, Western analysis was performed using anti-GFP antibody. No full-length PkaC1 was detectable in the strains lacking PKA activity (Fig. 3B), suggesting transcriptional repression or posttranslational degradation of the mutant proteins* in vivo*. Comparison of PkaC1 transcript levels between the mutant strains and wild-type control using a quantitative reverse transcription-PCR (RT-PCR) approach revealed no significant differences in transcriptional activity (Fig. S3), suggesting downregulation of the protein at a posttranslational level. To overcome this issue, we then overexpressed the PkaC1-T331E, PkaC1-T333A, and PkaC1-T337E mutant forms (representing the predicted inhibitory phosphorylation states at each site) using a constitutively active *otef* promoter (26) to verify their activities in comparison to the wild-type PkaC1. Western blotting revealed comparable levels of the relevant full-length protein in each of these strains (Fig. 3C, upper panel), but PKA enzymatic activity as measured by its ability to phosphorylate the fluorescent peptide was still undetectable in extracts of the strains expressing the mutant isoforms (Fig. 3C, lower panel). This suggested that, although the full-length mutant proteins were abundant, their enzymatic activity was dramatically compromised by the mutations within the activation segment. Taken together, these results are in agreement with predictions made based on our structural modeling of PkaC1, indicating the potential for severe disruption of PKA activity resulting from substitutions in the activation segment of PkaC1, while substitutions in the vicinity of S175 would not have been expected to significantly affect enzyme activity.

10.1128/mBio.02319-16.4 FIG S3  Transcription of PkaC1 phosphomutant isoform genes. Total RNA was extracted from 24-h liquid cultures of the indicated strains, followed by cDNA synthesis using oligo(dT) primers. Quantitative real-time PCR (qPCR) was then performed in order to amplify PkaC1 cDNA as well as β-tubulin cDNA as a control. Three replicate qPCRs were performed for each sample. Columns represent the average proportion of the WT cDNA level for each set of replicates as determined by subtraction of control β-tubulin *C*_*T*_ values from corresponding PkaC1 *C*_*T*_ values, followed by subtraction of these values from the average of the WT samples. 2 was then raised to this calculated value to determine the proportion of WT represented by each. Bars represent the averages of these values for the three replicates for each strain. Error bars represent standard errors. No statistically significant differences were identified between samples, with the exception of the Δ*pkaC1* mutant, which produced no measurable PkaC1 cDNA and was found to be significantly different from each of the other samples (*P* < 0.003). Download FIG S3, PDF file, 0.3 MB. Copyright © 2017 Shwab et al. 2017 Shwab et al. This content is distributed under the terms of the Creative Commons Attribution 4.0 International license.

### Binding of the PkaC1 catalytic subunit to the PkaR regulatory subunit is not eliminated by the T333A mutation in PkaC1.

It is well established that the binding of the PKA catalytic subunit with the regulatory subunit is important for its activation and necessary for proper regulation of PKA signaling by cAMP in response to environmental signals (8, 25). As shown in Fig. 2B, the interaction between the two subunits is predicted to approximately mimic that of the interaction between the catalytic subunit and substrate peptides. Considering the possibility of the substitution mutations impacting the binding of PkaC1 to the PkaR regulatory subunit and inhibiting the activity of PKA, it was of interest to examine this interaction in the presence of the T333A mutation. To accomplish this, red fluorescent protein (RFP)-labeled PkaR (RFP-PkaR) and GFP-labeled PkaC1 isoform-expressing strains were generated with either the native PkaC1 sequence (PkaC1-GFP) or the T333A substitution (PkaC1-T333A-GFP) expressed from the constitutive *otef* promoter. Normalized protein extracts from each of these strains were then divided and subjected to GFP-Trap and RFP-Trap affinity purifications to isolate GFP-labeled PkaC1 isoforms and RFP-PkaR, respectively. Western analysis of the purified fractions with anti-GFP antibodies revealed the presence of PkaC1-GFP and PkaC1-T333A-GFP isolated from both GFP- and RFP-Trap-purified samples for the respective strains (Fig. S4). The presence of a faint band representing PkaC1-T333A-GFP in the RFP-Trap-purified extract from the mutant strain indicates that some degree of interaction occurs between the subunits in this mutant strain. The low intensity of the relevant band may suggest impairment in the interaction between the PkaR regulatory subunit and the mutated PkaC1 subunit, in keeping with observations from other fungal species (16–19). However, because the GFP-Trap-purified extracts from the PkaC1-GFP- and PkaC1-T333A-GFP-expressing strains contain varying amounts of the PkaC1 subunit, the observed differences may to some degree be attributed to differing expression levels of PkaC1 in these two strains in addition to reduced interaction between the PkaC1 and PkaR subunits.

10.1128/mBio.02319-16.5 FIG S4  Impact of PkaC1 mutation on regulatory subunit interaction. Extracts from strains expressing RFP-labeled PkaR and GFP-labeled PkaC1 of either the WT or T333A mutant sequence expressed via the constitutive *otef* promoter were subjected to both GFP-Trap and RFP-Trap affinity purification and then probed via Western blotting with anti-GFP antibodies as described above. GFP-Trap samples confirm the presence of full-length PkaC1 in each extract, and RFP-Trap samples indicate that PkaC1 was purified through association with PkaR in each sample, although considerably less protein was detected in the T333A mutant extract. Download FIG S4, PDF file, 0.1 MB. Copyright © 2017 Shwab et al. 2017 Shwab et al. This content is distributed under the terms of the Creative Commons Attribution 4.0 International license.

### Mutation of phosphorylated residues in PkaC1 causes pleiotropic effects on germination, growth, conidiation, and stress response of *A. fumigatus*.

**FIG 4:**
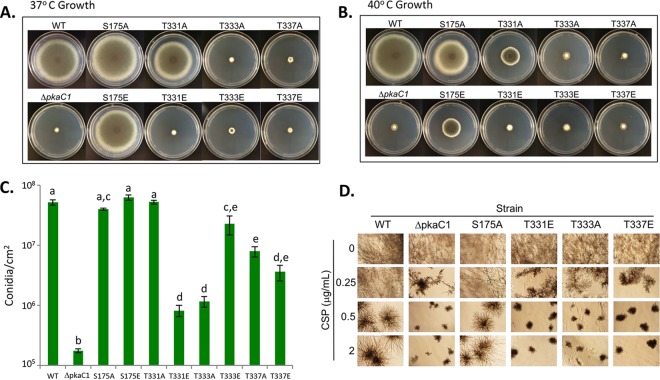
Growth, conidiation, and cell wall stress tolerance of *A. fumigatus pkaC1* mutants. (A and B) Conidia of the indicated strains were point inoculated onto GMM agar plates and incubated for 5 days at 37°C (A) or 40°C (B). T331E, T333A, T333E, T337A, and T337E strains showed marked radial growth defects at both temperatures, while defects were prominent only at 40°C for S175A, S175E, and T331A strains. (C) Quantitation of conidiation following harvest after 120 h of growth on GMM at 37°C. The T331E and T333A substitution mutants showed dramatically reduced conidiation, while all other mutants had wild-type levels or only moderately reduced conidiation. Columns represent averages from three replicates, and error bars represent standard errors. Different letters above columns represent statistically significant differences as determined by Student’s *t* test (*P* < 0.05). (D) Sensitivity of WT and Δ*pkaC1* mutants to caspofungin (CSP). Conidia (10^2^) were inoculated into liquid RPMI medium containing indicated concentrations of CSP and incubated at 37°C for 48 h. Loss of PKA in the deletion strain leads to more strongly inhibited growth at 0.25 µg/ml CSP and above, as do the T331E, T333A, and T337E mutations, while the S175A mutation has no visible effect on CSP sensitivity.

Considering the importance of phosphorylation of the residues in the activation segment of PkaC1 for its activity, we next assessed the functional importance of PkaC1 phosphorylation at the four residues for various aspects of growth, including germination, hyphal growth, conidiation, and tolerance to higher temperature and cell wall stress induced by the β-glucan-synthase-targeting antifungal caspofungin (CSP). Conidial germination, radial growth, and conidiation of these mutant strains were assessed in comparison to a strain expressing the wild-type (WT) protein and a Δ*pkaC1* strain lacking the gene encoding PkaC1. As shown in Fig. S5A, the T331E and T333A mutant strains had lower germination rates similar to that of the Δ*pkaC1* strain at the end of 8 h (<10%). Radial growth on glucose minimal medium (GMM) was severely impaired for several of the mutant strains (Fig. 4A and B and S5B). Substitution of alanine or glutamate for S175 (S175A and S175E, respectively) did not appear to affect radial growth at 37°C (Fig. 4A and S5B), but significant reduction in radial growth was noted at the elevated temperature of 40°C, with attenuation of growth being more pronounced in the S175E than in the S175A strain (Fig. 4B and S5B). Substitution of alanine for T331 (T331A) also produced a significant radial growth defect at 40°C (Fig. 4B and S5B). Substitution of glutamate at T331 (T331E), and substitutions of either alanine or glutamate at T333 and T337 (T333A, T333E, T337A, and T337E, respectively) all displayed highly attenuated radial growth similar to that of the Δ*pkaC1* strain.

10.1128/mBio.02319-16.6 FIG S5  Conidial germination, growth on osmotic stabilizing medium, and radial growth quantification of PkaC1 mutant strains. (A) Conidia (100) from the WT and the various *pkaC1* mutant strains were inoculated into 5 ml GMM liquid medium, and germination of conidia was quantified after conidia were incubated at 37°C and examined microscopically at 8 (light blue) and 24 (dark blue) hours to determine the proportions of germinated and ungerminated spores. Germination was strongly inhibited in the T331E and T333A substitution mutants as in the deletion strain, while all other substitution mutants had wild-type levels or only somewhat delayed germination rates. (B) Plates showing growth on OSM at 37°C. Conidiation was qualitatively rescued in deficient mutants by the presence of 1.2 M sorbitol. (C) Quantitation of mean radial growth diameters for each strain and growth condition is presented. Blue represents growth on GMM at 37°C, purple represents growth on GMM at 40°C, and green represents growth on OSM at 37°C. For all graphs, error bars represent 1 standard deviation and different letters above columns indicate statistical differences between strains at a *P* value of <0.05 based on Student’s *t* tests. Mutants with mutations at S175 displayed significant radial growth defects only at 40°C, while mutants with mutations at T331, T333, and T337 showed significant defects under all conditions. Growth rates of mutants with strongly attenuated growth on GMM were significantly increased on OSM. Agar plates were point inoculated with 10^4^ conidia of the indicated strains and incubated at the indicated temperatures for 120 h. Assays were performed in triplicate, and a representative plate is shown for each strain and condition. Download FIG S5, PDF file, 0.5 MB. Copyright © 2017 Shwab et al. 2017 Shwab et al. This content is distributed under the terms of the Creative Commons Attribution 4.0 International license.

Severe reduction in conidiation was also observed on GMM in the T331E and T333A mutant strains in comparison to the WT strain, while more moderate reductions were observed for T333E, T337A, and T337E (Fig. 4C). However, in each case, conidiation was not as sparse as that observed for the Δ*pkaC1* strain.

It was previously noted that the conidiation defect of the Δ*pkaC1* strain can be rescued on osmotically stabilizing medium (OSM) supplemented with sorbitol (9). To determine if this was true for the PkaC1 phosphorylation mutant strains, the strains were also grown on OSM. As in the case of the Δ*pkaC1* strain, all strains with conidiation defects demonstrated macroscopically observable increases in conidiation on OSM, in addition to significantly increased radial growth (Fig. S5B and S5C), suggesting that the impairment in conidiation may involve defects in cell wall integrity which are ameliorated by osmotic stabilization. PKA has also been shown to contribute to the tolerance of *A. fumigatus* to the cell wall-damaging agent Congo red (9). In order to determine whether or not the phosphorylation state of PkaC1 can influence *A. fumigatus* cell wall integrity, and in order to assess the role of PKA signaling in promoting tolerance to a medically important antifungal agent, the various PkaC1 phosphomutant strains along with the WT and Δ*pkaC1* strains were cultured in the presence of various concentrations of CSP and examined via microscopy (subset of strains and CSP concentrations shown in Fig. 4D and full set shown in Fig. S6). At concentrations of 0.5 to 4 µg/ml CSP, the WT strain exhibited an aberrant but still prolific growth phenotype, while growth of the Δ*pkaC1* strain became aberrant at the lower CSP concentration of 0.25 µg/ml and was more severely attenuated than that of the WT at higher levels. Among the strains expressing mutant PkaC1, the S175A, S175E, and T331A mutants displayed growth very similar to that of the WT strain, while growth of the T331E mutant and both mutants at T333 and T337 closely resembled the attenuated phenotype of the Δ*pkaC1* strain. Notably, the “paradoxical effect” noted for wild-type *A. fumigatus*, in which increased hyphal growth occurs in the presence of higher concentrations of CSP (27), observable here as increased filamentation of the WT, S175A, S175E, and T331A strains at 2 and 4 µg/ml CSP (compared to more restricted growth at 0.5 and 1 µg/ml), was not observed for the Δ*pkaC1* strain nor the T331E, T333A, T333E, T337A, or T337E mutants. To some extent, the T333E mutant showed slightly larger colony morphology at all the concentrations of CSP, which may relate to residual PKA activity (Fig. S6).

10.1128/mBio.02319-16.7 FIG S6  Sensitivity of PkaC1 mutants to caspofungin (CSP). Conidia (10^2^) were inoculated into liquid RPMI medium containing the indicated concentrations of CSP and incubated at 37°C for 48 h. Loss of PKA in the deletion strain leads to more strongly inhibited growth at 0.25 µg/ml CSP and above, as do the T331E, T333A, T333E, T337A, and T337E mutations, while the S175A, S175E, and T331A mutations have no visible effect on CSP sensitivity. Paradoxically increased growth was observed at 2 and 4 µg/ml CSP for the WT and the S175A, S175E, and T331A mutants only. Download FIG S6, PDF file, 0.5 MB. Copyright © 2017 Shwab et al. 2017 Shwab et al. This content is distributed under the terms of the Creative Commons Attribution 4.0 International license.

### Mutation of phosphorylated residue T333 of PkaC1 causes attenuated virulence of *A. fumigatus*.

**FIG 5:**
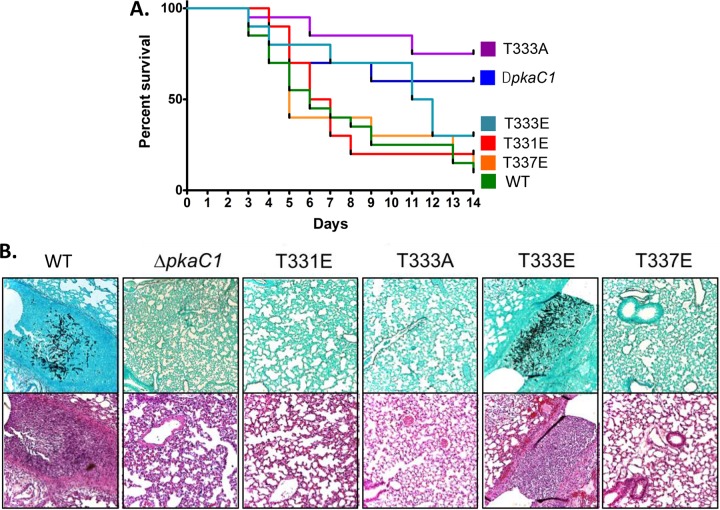
Virulence of *A. fumigatus* mutants in a murine infection model. (A) Survival of mice infected with mutant strains was plotted using Kaplan-Meier curves and analyzed using a log rank pairwise comparison (*P* < 0.05). A total inoculum of 4 × 10^6^ conidia was delivered intranasally to mice immunosuppressed with cyclophosphamide and triamcinolone acetonide, and survival was scored daily for 14 days. Mortality caused by all mutant strains with the exception of the T333A strain was not statistically different from that with the WT control strain, while the T333A strain was similar to the Δ*pkaC1* strain. (B) Lung histopathologic examination at 3 days postinfection. Gomori’s methenamine silver staining (top row) showed appreciable hyphal growth only in the animals infected with the PkaC1-GFP positive-control strain and the T333E mutant strain. Hematoxylin and eosin staining (bottom row) showed inflammation and tissue damage in these same strains. Tissue infected with the other mutant strains displayed the same lack of hyphal growth and tissue damage as that observed in the Δ*pkaC1* strain-infected negative control. Magnification, ×10.

The requirement of PKA for virulence of *A. fumigatus* was previously demonstrated by the deletion of *pkaC1*, which led to attenuated virulence; complete abolition of virulence occurred in a strain harboring the deletion of both the catalytic isoforms *pkaC1* and *pkaC2* (9). Based on our structural modeling and the phenotypic data for the various phosphorylation mutants of PkaC1, we investigated how these mutations impacted virulence. Virulence was examined first in the invertebrate wax moth larva (*Galleria mellonella*) model, followed by a persistently neutropenic murine model of IA. In *G. mellonella*, virulence of each alanine and glutamate substitution strain was compared to that of the WT and Δ*pkaC1* strains as controls (Fig. S7). Patterns of virulence among the mutants in the *G. mellonella* model were similar to those observed for radial growth, with mortality rates from the T331E, T333A, T333E, T337A, and T337E mutants being significantly lower than that caused by infection with the WT control strain and not significantly different from that of the Δ*pkaC1* strain. Following these virulence screening assays in* Galleria*, a subset of mutant strains was tested in the persistently neutropenic murine model of IA. Mice were infected intranasally with the WT, Δ*pkaC1*, T331E, T333A, T333E, and T337E strains, with the first two strains serving as controls. The T331E and T337E mutants were selected as mimicking the predicted inhibitory phosphorylation states of the enzyme at these sites, based on the invertebrate model infection screening, observed radial growth and conidiation, and structural modeling. Both the alanine and glutamate T333 substitution strains were included, as the T333A mutant is predicted to mimic the inhibited, dephosphorylated state of the enzyme while T333E should more closely resemble the active, phosphorylated state. While both the T333A and T333E mutants showed reduced virulence in the *G. mellonella* model, we were interested in testing the T333E mutant as well. As noted, this strain displayed increased conidiation and germination rates compared to the T333A mutant. Combined with predictions from structural modeling, these results suggest the possibility of somewhat increased PKA activity of this mutant compared to the T333A mutant. Despite all of the substitution mutant strains tested displaying radial growth and *G. mellonella* virulence phenotypes similar to those of the Δ*pkaC1* strain, only the T333A strain showed significantly reduced virulence in the murine model compared to the WT control strain (Fig. 5A). This suggests that PKA activity may be more strongly attenuated in this mutant than the others and that a low level of PKA activity is sufficient to maintain virulence. However, histological examination of lung tissue from mice infected with each of these strains revealed a lack of hyphal growth and alveolar damage/inflammation 3 days postinfection for all strains except for the WT positive control and the T333E mutant (Fig. 5B), suggesting that despite similar outcomes in terms of eventual mortality, mutation of PkaC1 to mimic inactive phosphorylation states results in attenuated pathogenicity during the early stages of infection.

10.1128/mBio.02319-16.8 FIG S7  Virulence of *A. fumigatus* mutants in *Galleria mellonella* infection model. Survival of *G. mellonella* larvae infected with mutant strains was plotted using Kaplan-Meier curves and analyzed using log rank pairwise comparison (*P* < 0.05). Virulence of alanine (A) and glutamate (B) substitution mutants in *G. mellonella*. Larvae were inoculated with 2 × 10^5^ conidia and incubated at 37°C, and survival was scored daily for 5 days. S175A, S175E, and T331A mutants showed wild-type levels of virulence, while all other mutants had reduced virulence statistically similar to infection with the Δ*pkaC1* strain. Download FIG S7, PDF file, 0.7 MB. Copyright © 2017 Shwab et al. 2017 Shwab et al. This content is distributed under the terms of the Creative Commons Attribution 4.0 International license.

## DISCUSSION

**FIG 6:**
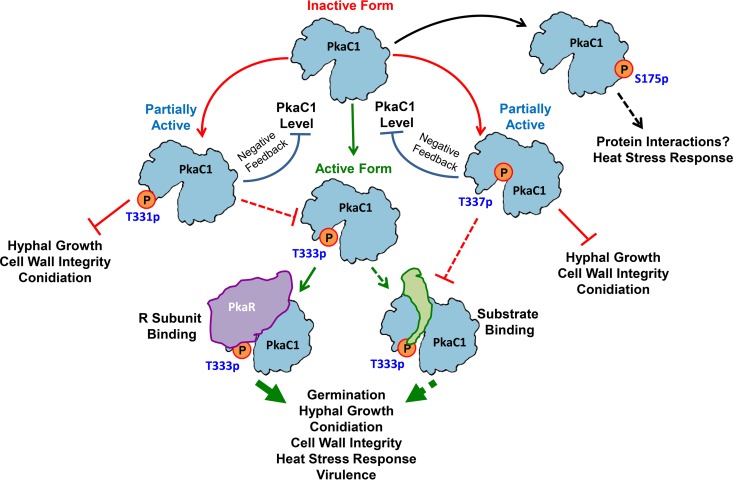
Hypothetical model depicting the effect of phosphorylation of PkaC1 at different residues on its activity and function in *A. fumigatus*. Phosphorylation at T333 leads to activation of PkaC1 via stabilization of substrate and regulatory subunit binding, promoting hyphal growth, conidiation and germination, cell wall integrity, heat stress tolerance, and virulence. In contrast, phosphorylation at T331 or T337 destabilizes substrate binding, causing partial inactivation of PkaC1. In the case of T337 phosphorylation, this likely occurs via blockage of T333 phosphorylation, while T337 phosphorylation is expected to directly inhibit substrate binding due to steric hindrance. As a result, T331 and T337 phosphorylation both lead to inhibition of hyphal growth, conidiation, and cell wall integrity. This suppression of PKA activity also leads to downregulation of PkaC1 protein levels in a negative-feedback mechanism. A dynamic phosphorylation state at S175 is necessary for mounting a normal heat stress response, possibly due to a role for this site in particular unknown protein-protein interactions. Solid lines represent experimentally validated findings, while dashed lines represent hypothesized processes.

In this study, we have identified a novel mechanism for the regulation of PKA activity in* A. fumigatus*, a leading fungal pathogen of immunocompromised patients. While PKA activation via phosphorylation of the T-loop residue is known to occur in other species, this is the first report on negative phosphoregulation of PKA in any system. In contrast to mammalian PKA, we have shown that in *A. fumigatus* the activation segment of the PKA catalytic subunit may be used as a molecular switch, promoting or suppressing enzyme activity depending on the phosphorylation state of specific key residues in the loop (Fig. 6). Although very little is known about PKA signaling in filamentous fungi, it is possible that this phosphoregulatory mechanism is common in this group. Evidence for additional, unidentified phosphorylation sites besides the T-loop residue in diverse yeast species (16, 20) also leaves open the possibility that expanded phosphoregulation of PKA activity may be a shared feature of fungi, which could represent an important distinction between fungal and mammalian PKA signaling.

In close agreement with our structural predictions by molecular modeling of *A. fumigatus* PkaC1, mutations mimicking altered phosphorylation at the four identified sites revealed striking effects on PKA activity and therefore the physiology of *A. fumigatus*. Phosphorylation at the T333 residue is expected to stabilize the substrate and facilitate regulatory subunit binding (Fig. 6), as was previously demonstrated for mammalian and *S. cerevisiae* PKA isoforms (14–16, 19), and indeed alanine substitution at this site resulted in pronounced phenotypic effects. In addition to phenotypic effects closely resembling those of the Δ*pkaC1* strain, regulatory subunit binding affinity appeared to be reduced by the T333A substitution as predicted by structural modeling, though our evidence suggests that association between the subunits does still occur to some degree in the presence of this mutation. This is in accordance with findings in *S. cerevisiae*, wherein R-subunit binding and inhibition were reduced but not eliminated by loss of T-loop residue phosphorylation (16, 19). The observed phenotypic defects of the T333E phosphomimetic mutant strain appear to contrast with findings from other fungi, wherein strains with glutamate and aspartate substitutions for the corresponding T-loop residues were able to more closely phenocopy the wild-type strain (16, 17). This indicates the possibility that, unlike in other fungi, glutamate is inadequate to biochemically mimic phosphothreonine at this site within the *A. fumigatus* enzyme and/or that dynamic phosphorylation and dephosphorylation of the T-loop residue is necessary for fully effective PKA signaling in this species.

Structural modeling predicted inhibitory roles for phosphorylation at the remaining two identified activation segment residues, T331 and T337, in that T331 phosphorylation would likely block that of the adjacent T333 and might otherwise destabilize substrate binding, while the location of T337 near the site of catalysis would mean that phosphorylation at this site could potentially disrupt both substrate binding and phosphate transfer (Fig. 6). Accordingly, the T331E substitution mutant had much more pronounced phenotypic defects than did the T331A mutant and indeed closely resembled the T333A mutant in all attributes examined with the exception of murine virulence. This is consistent with the hypothesis that the primary function of T331 phosphorylation would be to preclude that of T333, thus inhibiting enzyme activity indirectly. However, the increased virulence in murine infection observed for the T331E strain compared to the T333A strain suggests that PKA signaling is not entirely suppressed in this mutant. Given the close proximity of the two residues, it is conceivable that T331 phosphorylation on its own might serve to stabilize substrate binding, though to a much lesser degree than that of phosphorylation at T333, thus permitting low-level PKA activity. The more minor growth defects observed in the T331A substitution strain, particularly in response to increased temperature, may be an indication that the unphosphorylated threonine residue at this location plays an important but nonessential role in enzyme function that alanine cannot completely mimic. In the case of T337, replacement with either alanine or glutamate resulted in phenocopying the radial growth of Δ*pkaC1*, as might be expected given the predicted intimate association of this residue with the catalytic residues of the enzyme and the site of phosphate transfer. In recombinant mammalian PKA, replacement of the residue analogous to *A. fumigatus* T337 with alanine was found to abolish autophosphorylation at T197 and to result in a loss of catalytic activity (28). Thus, it seems likely that unphosphorylated threonine is required at this site for optimal catalytic activity. However, the fact that both T337 substitution mutants displayed higher conidiation and germination rates and that the T337E mutant had murine virulence similar to that of the wild-type strain suggests that perturbation of this site is less consequential for overall PKA activity than is the case for T333.

The greater murine virulence of the T333E, T331E, and T337E mutant strains than of the T333A strain is interesting given that none of these strains showed detectable PKA activity in our kinase assay, suggesting that a small amount of PKA activity may be sufficient for virulence in a mammalian host. This concept is supported by the fact that, despite the apparently very low contribution of the secondary catalytic subunit (PkaC2) to overall PKA signaling, PkaC2 expression alone is sufficient for moderate virulence in murine infection (9). The increased conidiation and germination of the T333E and T337E strains compared to the Δ*pkaC1* and T333A strains suggest increased PKA activity in these strains, despite this being below the detection limits of our kinase assay, which may explain the increased virulence of these mutants. In the case of T331E, the level of PKA activity may be even lower but nonetheless sufficient to enable virulence in an immunocompromised murine host. Thus, our virulence data support the idea that phosphorylation at T331 or T337 suppresses PKA activity but does not eliminate it.

S175 was not expected to directly influence catalytic function based on its predicted position within the protein tertiary structure and indeed was not found to do so, based on the results of our PKA activity assay. It seems likely that the phosphorylation state of this residue may affect PKA signaling through interactions with other proteins, which might in turn affect targeting of the enzyme to specific substrates. The only notable physiological effect observed in mutants at this site was a reduced tolerance to increased temperature, supporting a role for PKA signaling in regulating the *A. fumigatus* heat shock response, as noted in other fungal species (29–31). The fact that glutamate substitution at S175 produced a more pronounced growth defect than did alanine substitution suggests that phosphorylation at this residue may have an inhibitory effect on some interactions, or it is possible that temporary phosphorylation here may enhance some transient interactions that are beneficial only in the context of particular environmental conditions (e.g., heat stress), subcellular locations, etc., making constitutive phosphorylation undesirable (Fig. 6).

The severe reduction in the amount of PkaC1 protein present in extracts from several mutant strains may reflect a novel mechanism of negative feedback regulation of PKA signaling in *A. fumigatus* (Fig. 6). Comparison of PKA activities in strains overexpressing wild-type and mutant PkaC1 indicated that the T331E, T333A, and T337E mutations result in a loss of detectable enzymatic activity even in the presence of abundant full-length protein. This result indicates that loss of PKA function likely precedes the observed reduction in protein level. The loss of natively expressed PkaC1 protein from strains with these substitutions and the presence of transcript levels similar to those of the wild type suggest that this enzyme is downregulated at a posttranslational level in the absence of active PKA signaling (Fig. 6), possibly via targeting of the protein for degradation via posttranslational modification. This differs from *S. pombe*, wherein substitution of alanine at the T-loop residue does not result in a reduction in protein level despite an apparent loss of PKA activity (17). Additionally, in *S. cerevisiae*, attenuation of PKA signaling results in increased promoter activity of catalytic subunit isoforms (32). Thus, the apparent reduction of PkaC1 expression in *A. fumigatus* resulting from loss of PKA activity seems to contrast with regulatory mechanisms identified in other fungal species studied.

The findings presented here indicate greater versatility in the phosphoregulation of PKA in *A. fumigatus* than in that of other biological systems studied. Unlike in mammalian species, here phosphorylation serves as a means of both positive and negative regulation of PKA activity (Fig. 6). More work is needed to further elucidate both the upstream regulation and downstream effects of PKA signaling in filamentous fungi and to further characterize the distinctions between these processes in pathogenic fungi and mammals. As evidence from *S. cerevisiae* indicates an important role for phosphorylation of the PKA regulatory subunit in the control of PKA signaling in this species (33–35), it would be pertinent to examine this process in *A. fumigatus*, particularly in light of the high degree of phylogenetic variation associated with the regulatory subunit which should increase the likelihood of identifying novel phosphoregulatory mechanisms. Also of note is the observed increase in sensitivity of several PkaC1 mutants to caspofungin, suggesting an important role for PKA signaling in the maintenance of cell wall integrity in *A. fumigatus*. Identification of specific PKA targets mediating caspofungin tolerance would be of considerable value in the development of new antifungal agents to complement and enhance the efficacy of echinocandin antifungals in the treatment of IA. Taken together, our observations begin to reveal the potential novelty of PKA signaling in filamentous fungi and underscore the need for additional insight into this and other signaling networks in this understudied but medically, ecologically, and economically important group of organisms.

## MATERIALS AND METHODS

### Fluorescence microscopy.

Conidia (10^4^) of GFP-labeled strains were cultured on coverslips immersed in 10 ml of GMM broth with or without the indicated concentrations of CSP and incubated for 20 h at 37°C. Hyphae were visualized using an Axioskop 2 Plus microscope (Zeiss) equipped with AxioVision 4.6 imaging software.

### Protein extraction and purification.

*A. fumigatus* recombinant strains expressing wild-type or mutant forms of PkaC1-GFP fusion protein were cultured in GMM liquid medium at 250 rpm for 24 h at 37°C with or without 1 μg/ml CSP. Total cell lysate was obtained by homogenizing mycelia (1 g wet weight) using a mortar and pestle as previously described (36, 37). Total protein in the crude extracts was quantified by the Bradford method, and samples were normalized. GFP-Trap and RFP-Trap (Chromotek) affinity purifications were performed from crude extracts according to the manufacturer’s instructions as previously described (37).

### PkaC1 purification, phosphopeptide enrichment, and LC-MS/MS analysis.

Total cell lysates were extracted and normalized to contain 10 mg protein in each sample before GFP-Trap affinity purification (Chromotek) and processed for TiO_2_ phosphopeptide enrichment and mass spectrometry as previously described (36, 37). Proteolytic digestion was accomplished by the addition of 500 ng sequencing-grade trypsin (Promega) directly to the resin, with incubation at 37°C for 18 h. Peptides were subjected to phosphopeptide enrichment using a 10-μl GL Sciences TiO_2_ Spin Tip. The dried phosphopeptide-enriched samples were resuspended and subjected to chromatographic separation on a Waters NanoAcquity ultraperformance liquid chromatograph (UPLC) equipped with a 1.7-μm high-strength silica T3 C_18_ 75-μm-inside-diameter (i.d.) by 250-mm reversed-phase column. The analytical column was connected to a fused silica PicoTip emitter (New Objective, Cambridge, MA) with a 10-µm tip orifice. Non-phosphopeptide-enriched samples were analyzed on a Synapt G2 quadrupole time of flight (QTOF) mass spectrometer operating in a data-dependent mode of acquisition. Phosphopeptide-enriched samples were analyzed on a QExactive Plus mass spectrometer also using a data-dependent mode of acquisition. MS/MS spectra of the 10 most abundant precursor ions were acquired with a collision-induced dissociation (CID) energy setting of 27, and a dynamic exclusion of 20 s was employed for previously fragmented precursor ions.

### Qualitative identifications and selected ion chromatograms from raw LC-MS/MS data.

Raw LC-MS/MS data files were processed in Mascot Distiller (Matrix Science, Inc.) and then submitted to independent Mascot database searches (Matrix Science, Inc.) against a custom NCBI *Aspergillus* database containing both forward and reverse entries of each protein. Search tolerances were 5 or 10 ppm for precursor ions and 0.02 or 0.04 Da for product ions using trypsin specificity with up to two missed cleavages for phosphopeptide-enriched or non-phosphopeptide-enriched data, respectively. Carbamidomethylation (+57.0214 Da on C) was set as a fixed modification, whereas oxidation (+15.9949 Da on M), deamidation (+0.98 Da on N and Q), and phosphorylation (+79.98 Da on S, T, and Y) were considered variable modifications. All searched spectra were imported into Scaffold (Proteome Software), and scoring thresholds were set to achieve a protein false discovery rate of 0% using the PeptideProphet algorithm. Normalized spectral counts were used to estimate relative protein abundances for interaction studies. This was accomplished by adjusting the sum of the selected quantitative value for all proteins in the list within each MS sample to the average of sums of all MS samples present in the experiment. Only those proteins with at least two unique peptides to match were considered to be correct. Phosphorylation site localization was assessed by exporting peak lists directly from Scaffold into the online Ascore algorithm (http://ascore.med.harvard.edu/ascore.html).

### Modeling of PkaC1 three-dimensional structure.

The sequences of the *Aspergillus fumigatus* PKA catalytic subunit and PKA regulatory subunit were threaded using Coot (38) onto source models with the highest sequence homologies, *Saccharomyces cerevisiae* PKA (39) and *Mus musculus* PKR (40), respectively. For the purpose of examining the active site of the PKA enzyme, some amino acid residue side chains were manually adjusted to relieve obvious steric clashes. Structural figures were rendered in PyMOL (PyMOL Molecular Graphics System, version 1.8; Schrödinger, LLC).

### Construction of *pkaC1* mutations in *Aspergillus fumigatus*.

For detailed methodology, see Text S1 in the supplemental material. *Escherichia coli* DH5α competent cells were used for all subcloning experiments. Deletion of the gene encoding PkaC1 was accomplished by replacement of the *pkaC1* coding region with a selectable *pyrG* marker in an *akuB*^*KU80*^* pyrG*^−^ auxotrophic strain (see Table S1 for all strains used) via homologous recombination. Deletion was confirmed via PCR and Southern blotting. C-terminal GFP labeling of PkaC1 was accomplished by insertion of the PkaC1 coding region (lacking a stop codon) 5′ to, and in frame with, the enhanced GFP (EGFP) coding region within the pUCGH vector, followed by the insertion of a downstream flanking genomic DNA sequence. Linearized construct was transformed into the *akuB*^*KU80*^ strain, and transformants were identified via hygromycin B selection (150 μg ⋅ ml^−1^) as described previously (36). Transformants were verified for homologous integration by PCR and fluorescence microscopy. Recombinant strains were sequenced to confirm GFP labeling.

10.1128/mBio.02319-16.9 TEXT S1  Construction of *pkaC1* mutations in *Aspergillus fumigatus*. Expanded description of methodology used to generate mutant strains of *A. fumigatus* in this study. Download TEXT S1, DOCX file, 0.02 MB. Copyright © 2017 Shwab et al. 2017 Shwab et al. This content is distributed under the terms of the Creative Commons Attribution 4.0 International license.

Site-directed mutagenesis of the *pkaC1* coding region was accomplished by amplifying 5′ and 3′ regions of the gene overlapping at the points of mutation, using reverse primers for the 5′ regions and forward primers for the 3′ regions which were reverse complements of one another and contained appropriate base substitutions at the desired sites of mutagenesis. Fusion PCR was then performed for each mutation by adding both the 5′ and 3′ amplified fragments into the same reaction mixture, along with the forward primers for the 5′ sequences and reverse primers for the 3′ sequences (see Table S2 for all primer sequences), so as to generate full-length sequence containing the desired point mutations. These sequences were then inserted into plasmid pUCGH, followed by insertion of the same *pkaC1* downstream flanking sequence used for PkaC1-GFP labeling described above. Linearized plasmids were transformed into the *akuB*^*KU80*^ strain, followed by screening and verification as described above. Additionally, recombinant strains were sequenced to confirm *pkaC1* mutation and GFP fusion.

10.1128/mBio.02319-16.10 TABLE S2  Primers used in this study. Listing of PCR primers used in this study for molecular cloning and quantitative RT-PCR. Download TABLE S2, DOCX file, 0.02 MB. Copyright © 2017 Shwab et al. 2017 Shwab et al. This content is distributed under the terms of the Creative Commons Attribution 4.0 International license.

N-terminal RFP labeling of PkaR was accomplished by insertion of the PkaR coding region 3′ to, and in frame with, the RFP coding region (lacking a stop codon) of the pJW24-RFP vector, followed by the insertion of upstream and downstream genomic flanking sequences. Linearized construct was transformed into the *akuB*^*KU80*^* pyrG*^−^ auxotrophic strain, and selected transformants were screened for homologous recombination via PCR and DNA sequencing.

Ectopic overexpression of native PkaC1 and PkaC1 containing the T331E, T333A, and T337E point mutations was accomplished by amplification of the *pkaC1* coding region genomic DNA from strains AF293, PkaC1-T331E-GFP, PkaC1-T333A-GFP, and PkaC1-T337E-GFP, respectively (Table S1), followed by insertion of amplified fragments into pUCGH, immediately 3′ to the *otef* promoter region (26). Circular plasmid constructs were then used to transform the *akuB*^*KU80*^ strain, followed by hygromycin B selection and screening via fluorescence microscopy, PCR, and sequencing to confirm transformations as described above.

Combined expression of RFP-labeled PkaR with either GFP-labeled native PkaC1 or T333A-mutated PkaC1 was accomplished by transformation of strain RFP-PkaR (Table S1) with circular constructs as described above for ectopic expression of the two GFP-labeled isoforms of PkaC1. Screening of transformants was performed as described above.

### Western blot analysis.

For direct blotting of crude extracts, approximately 50 μg of total protein was transferred onto a polyvinylidene difluoride (PVDF) membrane (Bio-Rad) following SDS-PAGE electrophoresis and probed with rabbit polyclonal anti-GFP primary antibody and peroxidase-labeled anti-rabbit IgG antibody or mouse monoclonal anti-α-tubulin primary antibody and peroxidase-labeled anti-mouse IgG antibody followed by detection using SuperSignal West Pico chemiluminescent substrate (Thermo Scientific). Following GFP-Trap and RFP-Trap (Chromotek) affinity purifications, samples were incubated at 100°C for 5 min to denature proteins and cause dissociation from the resin prior to blotting.

### Determination of PKA enzyme activity.

Nine microliters of crude protein extract normalized by the Bradford method was added in duplicate to reaction mixtures containing fluorescent dye-coupled PepTag A1 peptide LRRASLG (Promega) as a test substrate and incubated for 30 min at 25°C. Activity was assessed qualitatively per the manufacturer’s instructions based on migration of negatively charged, phosphorylated peptide toward the anode of an agarose gel. A standard curve using a range of 0 to 18 ng purified recombinant PKA catalytic subunit (Promega) was generated to quantify activity. Gel fragments containing phosphorylated peptide were excised and melted, and fluorescence readings were taken using a 540-nm excitation wavelength in a 96-well plate using a SpectraMax M3 plate reader (Molecular Devices).

### Quantitative RT-PCR.

Total RNA was extracted from 24-h liquid cultures by grinding frozen tissue with a mortar and pestle followed by RNA purification using the RNeasy minikit (Qiagen). First-strand cDNA synthesis was performed using the Tetro cDNA synthesis kit (Bioline) in the presence of oligo(dT)_15_ primer according to the manufacturer’s instructions. PCR was performed using a Bio-Rad iCycler and the Sensi-fast SYBR and fluorescein kit (Bioline). All amplification reactions included an enzyme activation step at 95°C (10 min), 40 cycles of denaturation at 95°C (15 s), annealing at 55°C (15 s), and an extension at 72°C (15 s) followed by a melt curve to ensure consistent product identity. Reactions were performed in triplicate for each sample with cDNA-specific primers for PkaC1 (PkaC1-RT-F and PkaC1-RT-R) and β-tubulin (5′ β-tubulin and 3′ β-tubulin; see Table S2). Additional reverse-transcriptase-negative control reaction mixtures were included for each sample and target to ensure amplification of cDNA only. Relative PkaC1 cDNA levels were quantified by subtraction of control β-tubulin threshold cycle (*C*_*T*_) values from corresponding PkaC1 *C*_*T*_ values, followed by subtraction of these values from the average of the WT samples. 2 was then raised exponentially to this calculated value to determine the proportion of WT cDNA represented by each sample.

### Assessment of radial growth, conidiation, and CSP sensitivity.

For radial growth assays, conidia (10^4^) were point inoculated on GMM or OSM (consisting of GMM supplemented with 1.2 M sorbitol) and incubated for 5 days at 37°C or 40°C. Conidiation was quantified by harvesting conidia from GMM plates incubated for 5 days at 37°C in 0.05% Tween 80. Germination was quantified by microscopic counting of germinating conidia following 8 and 24 h of growth in liquid GMM at 37°C. The mean growth, conidiation, and germination rates for each of the strains were compared statistically by Student’s *t* test (*P* < 0.05). For CSP sensitivity determination, CLSI M38-A2 *in vitro* antifungal susceptibility standards were used (41).

### *Galleria mellonella* and murine invasive aspergillosis virulence assays.

Virulence of mutant strains was assessed in an iterative fashion using an invertebrate and then a murine model of invasive aspergillosis. For the initial invertebrate model, 20 larvae of the wax moth *G. mellonella* were infected with each of the PkaC1 alanine and glutamate substitution strains as well as *pkaC1-egfp* and Δ*pkaC1 A. fumigatus* strains, delivering 5 μl of a 1 × 10^8^-conidia/ml suspension. Infected larvae were incubated at 37°C, and survival was scored daily for 5 days (42). For the murine model, male mice (CD1; Charles River Laboratories, Raleigh, NC) were immunosuppressed with cyclophosphamide (175 mg/kg of body weight intraperitoneally, days −2 and +3) and triamcinolone acetonide (40 mg/kg subcutaneously, days −1 and +6). Forty microliters of 1 × 10^8^-conidia/ml suspensions of the relevant strains were delivered intranasally following a brief isoflurane anesthesia induction. Survival for both virulence models was plotted on Kaplan-Meier curves and analyzed using log rank pairwise comparison (*P* < 0.05). Animal studies were carried out in accordance with all of the guidelines of the Duke University Medical Center Institutional Animal Care and Use Committee (IACUC) and in compliance with the U.S. Animal Welfare Act (Public Law 98-198). The Duke University Medical Center IACUC approved all of the vertebrate studies. The studies were conducted in Division of Laboratory Animal Resources (DLAR) facilities that are accredited by the Association for Assessment and Accreditation of Laboratory Animal Care (AAALAC).

### Histopathological analysis.

To characterize *in vivo* disease histopathology, two additional mice were infected with each of the analyzed strains as described above. Mice were euthanized on day +3 after inoculation, and lungs were harvested. Lung sections were stained with Gomori’s methenamine silver stain to visualize fungal hyphae and with hematoxylin and eosin stains to examine inflammation and tissue damage (42).
