# Supplementary material for: A Novel Phosphoregulatory Switch Controls the Activity and Function of the Major Catalytic Subunit of Protein Kinase A in Aspergillus fumigatus
Source: mBio. 2017 Feb 7;8(1):e02319-16. doi: 10.1128/mBio.02319-16 (PMC5296607; doi:10.1128/mBio.02319-16)
Supplement: FIG S1 [file mbo001173178sf1.pdf]

Figure S1

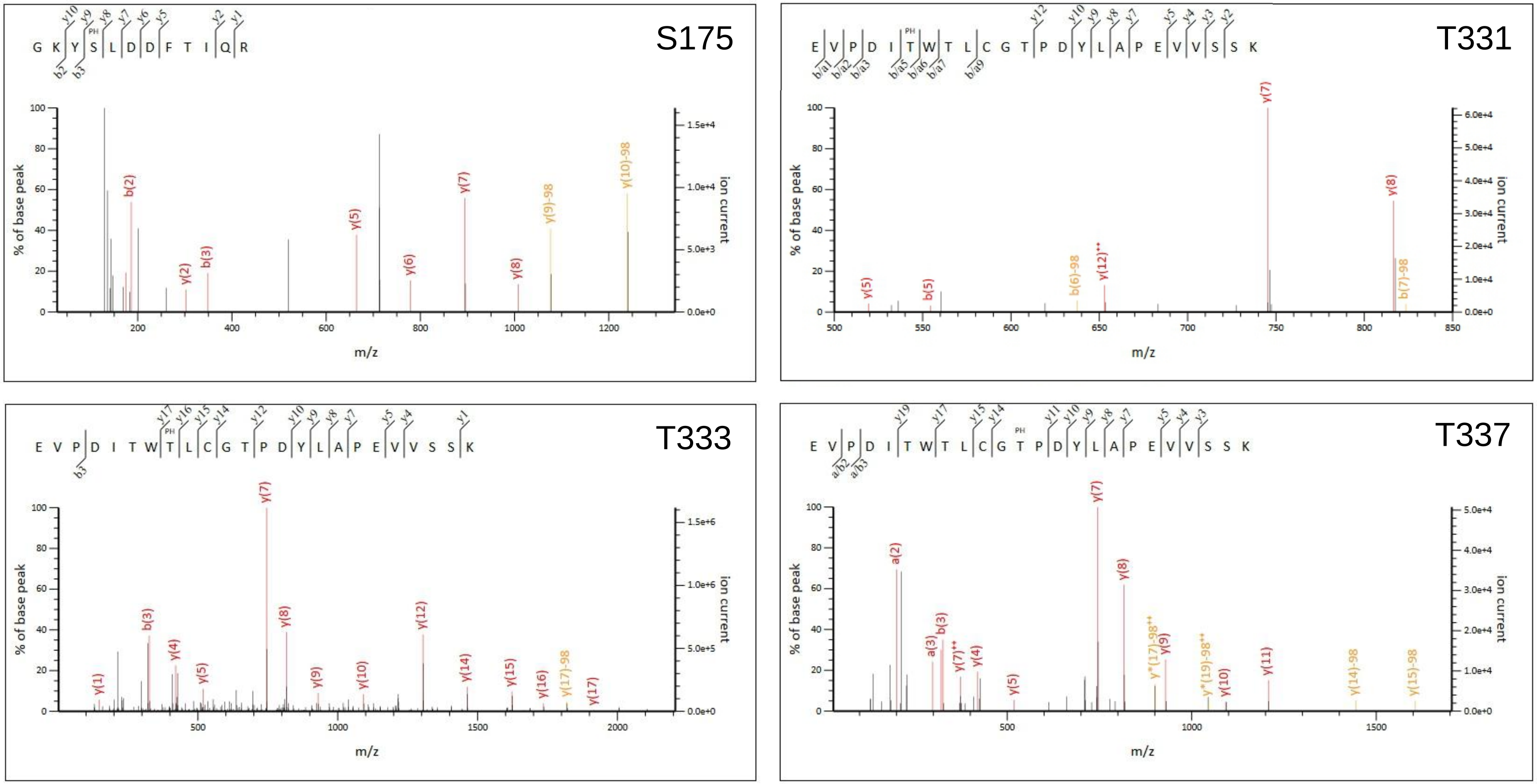

Figure S1. Spectra indicating phosphorylation at specific PkaC1 residues. Tandem mass spectra of GKY[pS]LDDFTIQR and EVPDI[pT]W[pT]LCG[pT]PDYLAP from PkaC1 reveal four unique phosphorylated residues including one serine (S175) and three threonines (T331, T333 and T337) in close proximity. The presence of each identified C-terminal (y) and N-terminal (b) product ions are indicated within the peptide sequence. Peaks indicating phosphorylation are represented in yellow.
