# Supplementary material for: A Novel Phosphoregulatory Switch Controls the Activity and Function of the Major Catalytic Subunit of Protein Kinase A in Aspergillus fumigatus
Source: mBio. 2017 Feb 7;8(1):e02319-16. doi: 10.1128/mBio.02319-16 (PMC5296607; doi:10.1128/mBio.02319-16)
Supplement: FIG S2 [file mbo001173178sf2.pdf]

Figure S2

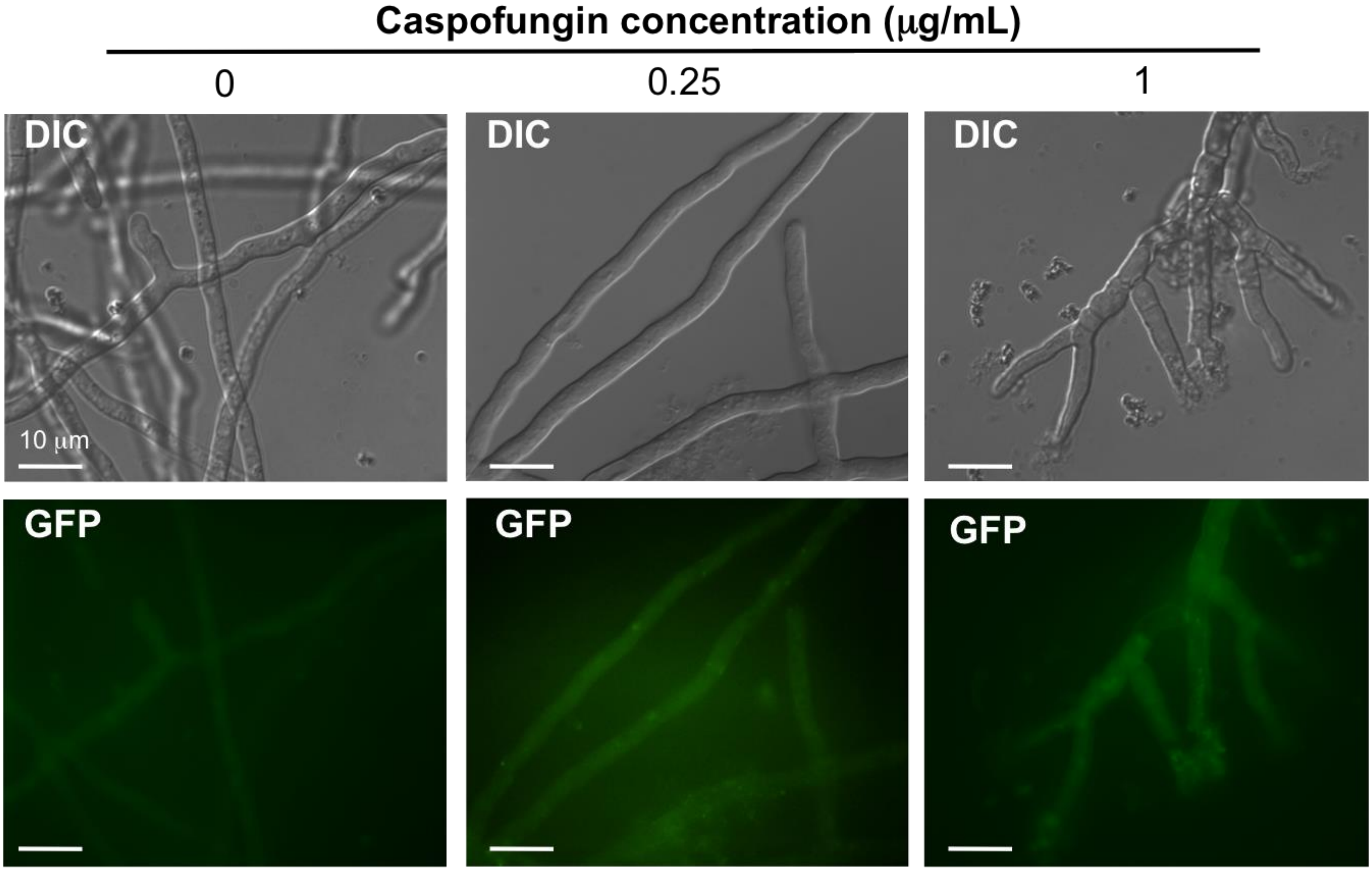
Caspofungin concentration (μg/mL)
0
0.25
1
DIC
DIC
DIC
10 μm
GFP
GFP
GFP

Figure S2. Localization of PkaC1 during caspofungin exposure. Conidia (10^4) of the strain expressing GFP-labeled PkaC1 (PkaC1-GFP) were cultured on coverslips immersed in GMM broth with or without the addition of the indicated concentrations of caspofungin (CSP) and incubated for 20 h at 37°C. Hyphae were visualized using an Axioskop 2 plus microscope (Zeiss) equipped with AxioVision 4.6 imaging software. Differential interference contrast (DIC) images are shown in grayscale, while GFP fluorescence is shown in green. In all samples, PkaC1 appears to be cytosolically localized.
