# Supplementary material for: A Novel Phosphoregulatory Switch Controls the Activity and Function of the Major Catalytic Subunit of Protein Kinase A in Aspergillus fumigatus
Source: mBio. 2017 Feb 7;8(1):e02319-16. doi: 10.1128/mBio.02319-16 (PMC5296607; doi:10.1128/mBio.02319-16)
Supplement: TABLE S1 [file mbo001173178st1.docx]

**SUPPLEMENTARY TABLES**

**Table S1. *A. fumigatus* strains used in this study**

| **Strain** | **Parent strain** | **Genotype** | **Reference** |
| --- | --- | --- | --- |
| *akuB^KU80^* | CEA17 | Wild-type | (1) |
| *akuB^KU80^* *pyrG*- | CEA17 *pyrG^+^* | *pyrG* | (1) |
| *ΔpkaC1* | *akuB^KU80^* *pyrG*- | *ΔpkaC1:: pyrG* | This study |
| *pkaC1*-*egfp*  (WT) | *akuB^KU80^* | *ΔpkaC1::pkaC1promo-pkaC1–egfp-hph* | This study |
| *pkaC1^mt^*-*S175A*-*egfp*  (S175A) | *akuB^KU80^* | *ΔpkaC1::pkaC1promo-pkaC1^mt^*-*S175A –egfp-hph* | This study |
| *pkaC1^mt^*-*S175E*-*egfp*  (S175E) | *akuB^KU80^* | *ΔpkaC1::pkaC1promo-pkaC1^mt^*-*S175E –egfp-hph* | This study |
| *pkaC1^mt^*-*T331A*-*egfp*  (T331A) | *akuB^KU80^* | *ΔpkaC1::pkaC1promo-pkaC1^mt^*-*T331A –egfp-hph* | This study |
| *pkaC1^mt^*-*T331E*-*egfp*  (T331E) | *akuB^KU80^* | *ΔpkaC1::pkaC1promo-pkaC1^mt^*-*T331E –egfp-hph* | This study |
| *pkaC1^mt^*-*T333A*-*egfp* (T333A) | *akuB^KU80^* | *ΔpkaC1::pkaC1promo-pkaC1^mt^*-*T333A –egfp-hph* | This study |
| *pkaC1^mt^*-*T333E*-*egfp*  (T333E) | *akuB^KU80^* | *ΔpkaC1::pkaC1promo-pkaC1^mt^*-*T333E –egfp-hph* | This study |
| *pkaC1^mt^*-*T337A*-*egfp*  (T337A) | *akuB^KU80^* | *ΔpkaC1::pkaC1promo-pkaC1^mt^*-*T337A –egfp-hph* | This study |
| *pkaC1^mt^*-*T337E*-*egfp*  (T337E) | *akuB^KU80^* | *ΔpkaC1::pkaC1promo-pkaC1^mt^*-*T337E –egfp-hph* | This study |
| *otef-pkaC1-egfp* (otef-WT) | *ΔpkaC1* | *ΔpkaC1 otefpromo-pkaC1-egfp-hph* | This study |
| *otef-pkaC1^mt^*-*T331E-egfp* (otef-T331E) | *ΔpkaC1* | *ΔpkaC1 otefpromo-pkaC1^mt^*-*T331E -egfp-hph* | This study |
| *otef-pkaC1^mt^*-*T333A-egfp* (otef-T333A) | *ΔpkaC1* | *ΔpkaC1 otefpromo-pkaC1^mt^*-*T333A -egfp-hph* | This study |
| *otef-pkaC1^mt^*-*T337E-egfp* (otef-T337E) | *ΔpkaC1* | *ΔpkaC1 otefpromo-pkaC1^mt^*-*T337E -egfp-hph* | This study |
| *rfp-pkaR* | *akuB^KU80^* *pyrG*- | *ΔpkaR::pkaRpromo-rfp-pkaR-pyrG* | This study |
| *otef-pkaC1-egfp rfp-pkaR* | *rfp-pkaR* | *otefpromo-pkaC1–egfp-hph ΔpkaR::pkaRpromo-rfp-pkaR-pyrG* | This study |
| *otef-pkaC1^mt^*-*T333A-egfp rfp-pkaR* | *rfp-pkaR* | *otefpromo-pkaC1^mt^*-*T333A –egfp-hph ΔpkaR::pkaRpromo-rfp-pkaR-pyrG* | This study |

1. **da Silva Ferreira ME, Kress MR, Savoldi M, M.H. G, Härtl A, Heinekamp T, Brakhage AA, Goldman GH.** 2006. The akuB(KU80) mutant deficient for nonhomologous end joining is a powerful tool for analyzing pathogenicity in Aspergillus fumigatus. . Eukaryot Cell **5:**207-211.
