# Supplementary material for: A Novel Phosphoregulatory Switch Controls the Activity and Function of the Major Catalytic Subunit of Protein Kinase A in Aspergillus fumigatus
Source: mBio. 2017 Feb 7;8(1):e02319-16. doi: 10.1128/mBio.02319-16 (PMC5296607; doi:10.1128/mBio.02319-16)
Supplement: FIG S3 [file mbo001173178sf3.pdf]

Figure S3

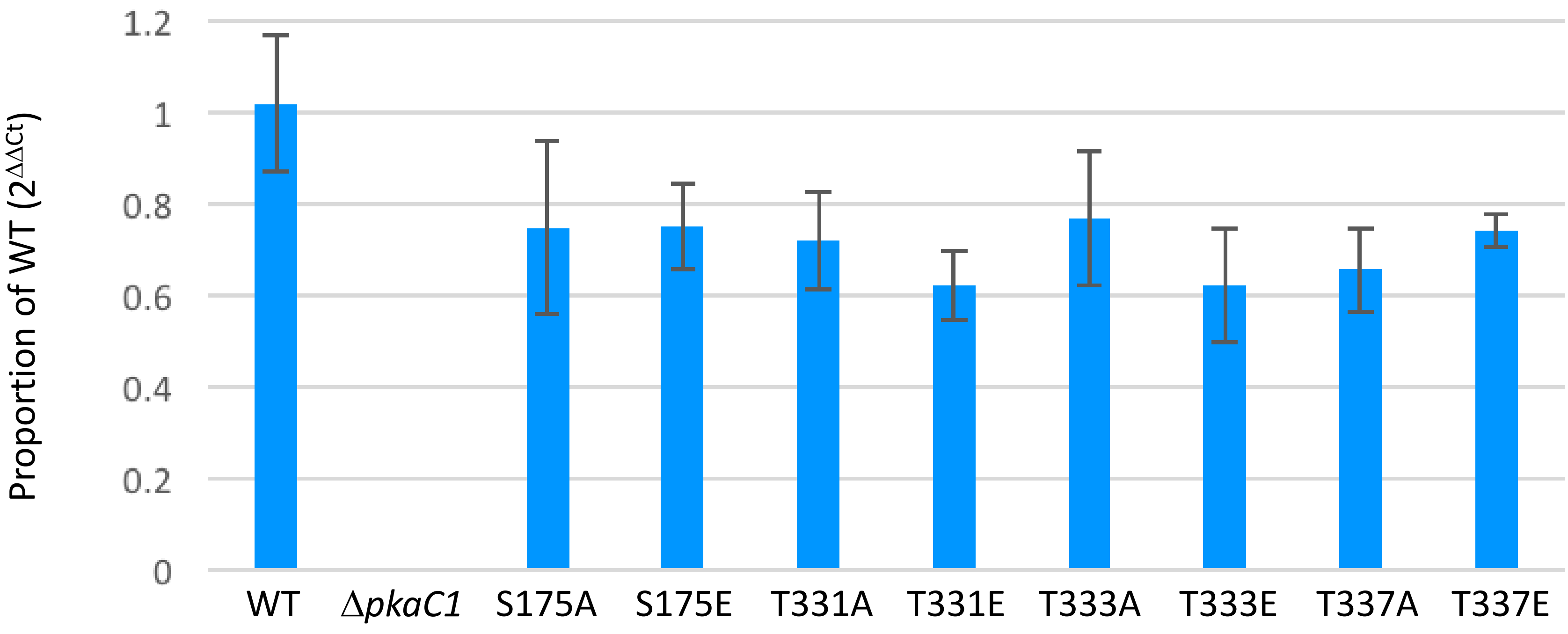

Figure S3. Transcription of PkaC1 phosphomutant isoform genes. Total RNA was extracted from 24 hour liquid cultures of the indicated strains, followed by cDNA synthesis using oligo dT primers. Quantitative real-time PCR (qPCR) was then performed in order to amplify PkaC1 cDNA as well as β-tubulin cDNA as a control. Three replicate qPCRs were performed for each sample. Columns represent the average proportion of the WT cDNA level for each set of replicates as determined by subtraction of control β-tubulin Ct values from corresponding PkaC1 Ct values, followed by subtraction of these values from the average of the WT samples. 2 was then raised to this calculated value to determine the proportion of WT represented by each. Bars represent the average of these values for the three replicates for each strain. Error bars represent standard error. No statistically significant differences were identified between samples, with the exception of Δ*pkaC1* which produced no measurable PkaC1 cDNA and was found to be significantly different from each of the other samples ($P < 0.003$).
