# Supplementary material for: A Novel Phosphoregulatory Switch Controls the Activity and Function of the Major Catalytic Subunit of Protein Kinase A in Aspergillus fumigatus
Source: mBio. 2017 Feb 7;8(1):e02319-16. doi: 10.1128/mBio.02319-16 (PMC5296607; doi:10.1128/mBio.02319-16)
Supplement: FIG S4 [file mbo001173178sf4.pdf]

Figure S4

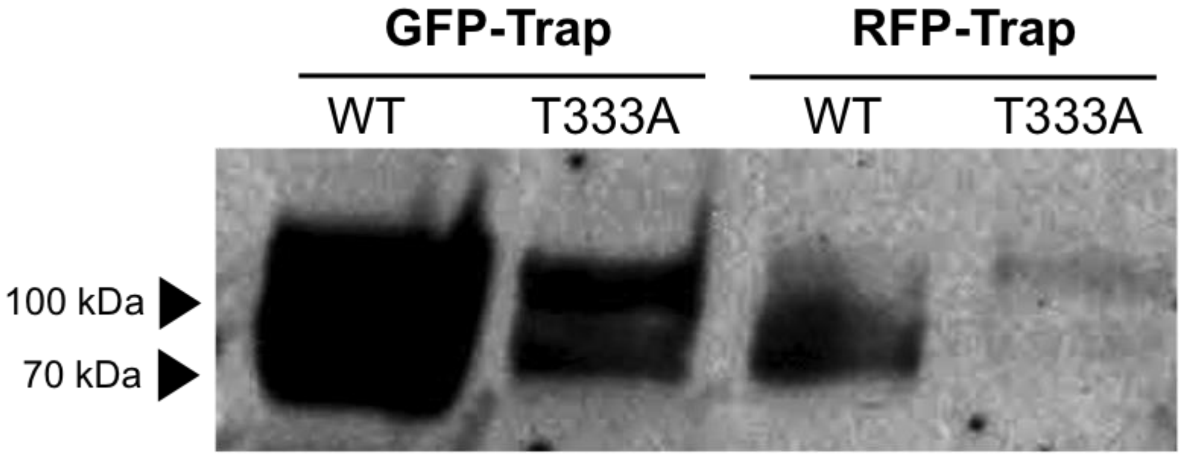

Figure S4. Impact of PkaC1 mutation on regulatory subunit interaction Extracts from strains expressing RFP-labeled PkaR and GFP-labeled PkaC1 of either the WT or T333A mutant sequence expressed via the constitutive *otef* promoter were subjected to both GFP-Trap® and RFP-Trap® affinity purification, then probed via Western blot with anti-GFP antibodies as above. GFP-Trap® samples confirm the presence of full length PkaC1 in each extract, and RFP-Trap® samples indicate that PkaC1 was purified through association with PkaR in each sample, though considerably less protein was detected in the T333A mutant extract.
