# Supplementary material for: A Novel Phosphoregulatory Switch Controls the Activity and Function of the Major Catalytic Subunit of Protein Kinase A in Aspergillus fumigatus
Source: mBio. 2017 Feb 7;8(1):e02319-16. doi: 10.1128/mBio.02319-16 (PMC5296607; doi:10.1128/mBio.02319-16)
Supplement: FIG S5 [file mbo001173178sf5.pdf]

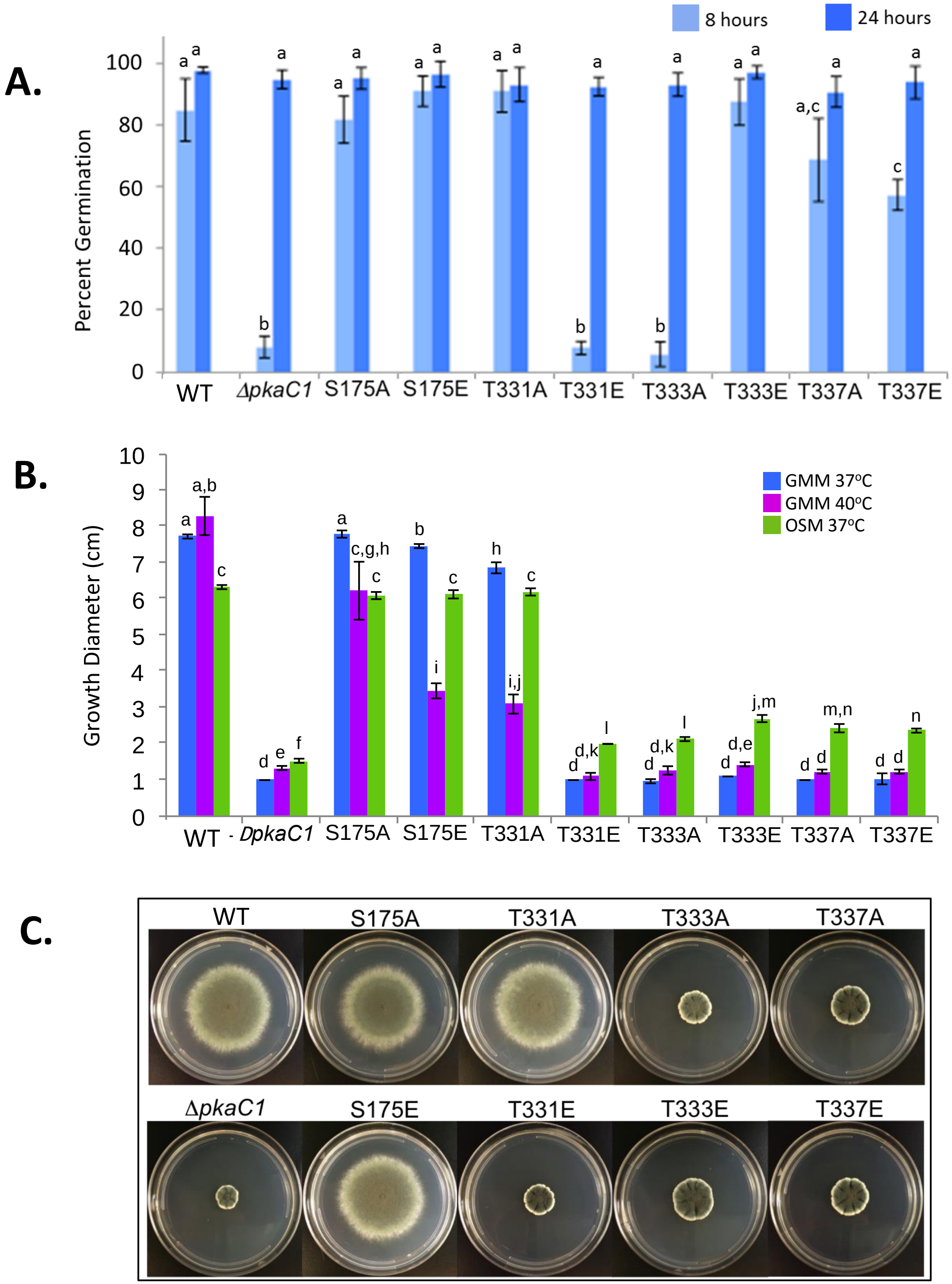
A.
8 hours
24 hours
Percent Germination
0
20
40
60
80
100
WT
ΔpkaC1
S175A
S175E
T331A
T331E
T333A
T333E
T337A
T337E
B.
Growth Diameter (cm)
GMM 37ºC
GMM 40ºC
OSM 37ºC
WT
DpkaC1
S175A
S175E
T331A
T331E
T333A
T333E
T337A
T337E
C.
WT
S175A
T331A
T333A
T337A
ΔpkaC1
S175E
T331E
T333E
T337E

Figure S5

Figure S5. Conidial germination, growth on osmotic stabilizing medium and radial growth quantification of PkaC1 mutant strains. (A) Conidia (100) from the WT and the various *pkaC1* mutant strains were inoculated into 5 ml GMM liquid medium and germination of conidia was quantified after conidia were incubated at 37˚C and examined microscopically at 8 (light blue) and 24 (dark blue) hours to determine the proportions of germinated and ungerminated spores. Germination was strongly inhibited in the T331E and T333A substitution mutants as in the deletion strain, while all other substitution mutants had wild-type level or only somewhat delayed germination rates. **(B)** Plates showing growth on OSM at 37˚C. Conidiation was qualitatively rescued in deficient mutants by the presence of 1.2M sorbitol. (**C**) Quantitation of mean radial growth diameters for each strain and growth condition is presented. Blue represents growth on GMM at 37˚C, Purple represents growth on GMM at 40˚C, and Green represents growth on OSM at 37˚C. For all graphs, error bars represent one standard deviation and different letters above columns indicate statistical differences between strains at $P < 0.05$ based on student's T-tests. Mutants at S175 displayed significant radial growth defects only at 40˚C, while mutants at T331, T333 and T337 showed significant defects under all conditions. Growth rates of mutants with strongly attenuated growth on GMM were significantly increased on OSM. Agar plates were point inoculated with 10^4 conidia of indicated strains and incubated at the indicated temperatures for 120 hours. Assays were performed in triplicate and a representative plate is shown for each strain and condition.
