# Supplementary material for: A Novel Phosphoregulatory Switch Controls the Activity and Function of the Major Catalytic Subunit of Protein Kinase A in Aspergillus fumigatus
Source: mBio. 2017 Feb 7;8(1):e02319-16. doi: 10.1128/mBio.02319-16 (PMC5296607; doi:10.1128/mBio.02319-16)
Supplement: FIG S6 [file mbo001173178sf6.pdf]

Figure S6

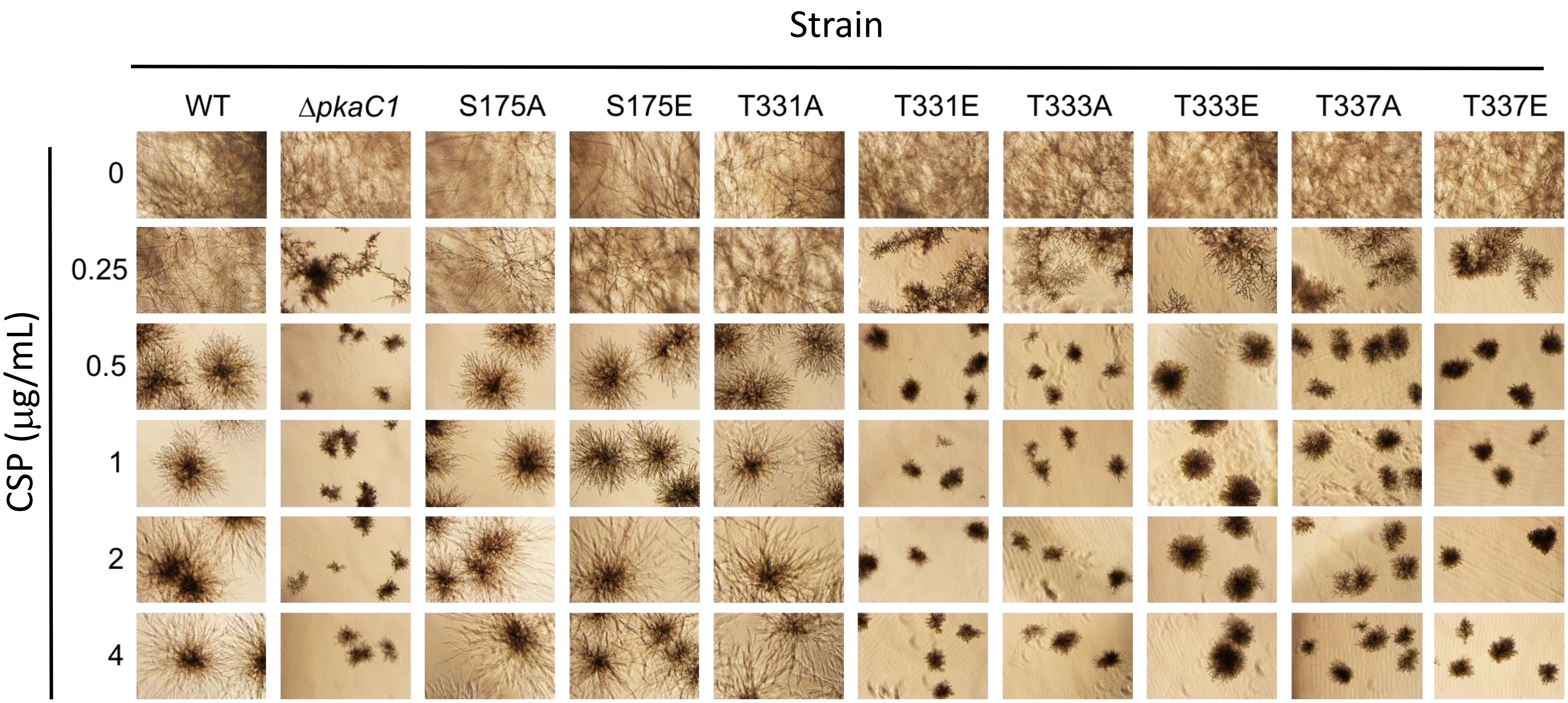

Figure S6. Growth, conidiation and cell wall stress tolerance of *A. fumigatus pkaC1* mutants. Sensitivity of WT and PkaC1 mutants to caspofungin (CSP). 10^2 conidia were inoculated into liquid RPMI medium containing 0, 0.5 or 1 µg/mL of CSP and incubated at 37˚C for 48 hours. Loss of PKA in the deletion strain leads to more strongly inhibited growth at 0.25 µg/mL CSP and above, as do the T331E, T333A, T333E, T337A and T337E mutations, while the S175A, S175E and T331A mutations have no visible effect on CSP sensitivity. Paradoxically increased growth was observed at 2 and 4 υg/mL CSP for the WT, S175A, S175E and T331A mutants only.
