# Supplementary material for: A Novel Phosphoregulatory Switch Controls the Activity and Function of the Major Catalytic Subunit of Protein Kinase A in Aspergillus fumigatus
Source: mBio. 2017 Feb 7;8(1):e02319-16. doi: 10.1128/mBio.02319-16 (PMC5296607; doi:10.1128/mBio.02319-16)
Supplement: FIG S7 [file mbo001173178sf7.pdf]

Figure S7

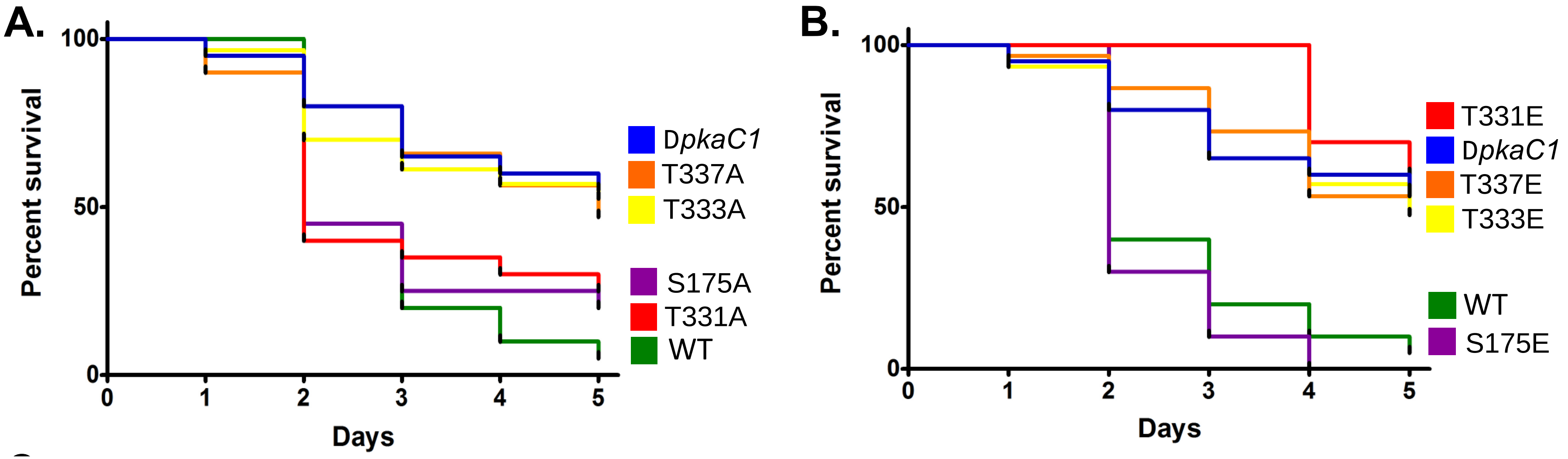

Figure S7. Virulence of *A. fumigatus* mutants in *Galleria mellonella* infection model. Survival of *G. mellonella larvae* infected with mutant strains was plotted using Kaplan–Meier curves and analyzed using log rank pair-wise comparison ($P < 0.05$). **(A, B)** Virulence of **(A)** alanine and **(B)** glutamate substitution mutants in *G. mellonella*. Larvae were inoculated with 2×10^5 conidia and incubated at 37°C and survival scored daily for 5 days. Mutants S175A, S175E and T331A showed wild-type level of virulence, while all other mutants had reduced virulence statistically similar to infection with the Δ*pkaC1* strain.
