# Supplementary material for: A Novel Phosphoregulatory Switch Controls the Activity and Function of the Major Catalytic Subunit of Protein Kinase A in Aspergillus fumigatus
Source: mBio. 2017 Feb 7;8(1):e02319-16. doi: 10.1128/mBio.02319-16 (PMC5296607; doi:10.1128/mBio.02319-16)
Supplement: TEXT S1 [file mbo001173178s1.docx]

**SUPPLEMENTAL MATERIAL**

**SUPPLEMENTARY METHODS**

**Supplementary Method S1. Construction of *pkaC1* mutations in *Aspergillus fumigatus***

*Escherichia coli* DH5α competent cells were used for all subcloning experiments. Deletion of the gene encoding PkaC1 was accomplished by insertion of approximately 1.6kb upstream and downstream flanking sequences amplified from AF293 genomic DNA (upstream using primers PkaC1-upstream-F(SalI) and PkaC1-upstream-R(EcoRI), downstream using primers PkaC1-downstream-F(NotI) and PkaC1-downstream-R(SacI), see Table S1 for all primer sequences) into SalI-EcoRI and NotI-SacI sites, respectively, on either side of the *pyrG* marker cassette within the pJW24 plasmid (1), followed by transformation of linearized construct into an *akuB^KU80^* *pyrG*- auxotrophic strain. Deletion was confirmed via PCR and Southern blot.

C-terminal GFP-labeling of PkaC1 was accomplished by insertion of the PkaC1 coding region (lacking a stop codon) amplified from AF293 genomic DNA (primers PkaC1-GFP-F(BamHI) and PkaC1-GFP-R(BamHI)) 5’ to - and in frame with - the *egfp* coding region into the BamHI site of the pUCGH vector, followed by the insertion of a downstream flanking sequence of about 900bp (amplified with primers PkaC1-downstream-F(SbfI) and PkaC1-downstream-R), into an SbfI-HindIII restriction site. Linearized construct was transformed into the *akuB^KU80^* strain and transformants were identified via hygromycin B selection (150 μg·mL^−1^) as described (2). Transformants were verified for homologous integration by PCR and fluorescence microscopy. Recombinant strains were sequenced to confirm GFP labeling.

Site-directed mutagenesis of the *pkaC1* coding region was accomplished by amplifying 5’ and 3’ regions of the gene overlapping at the points of mutation, using reverse primers for the 5’ regions and forward primers for the 3’ regions which were reverse complements of one another and which contained appropriate base substitutions at the desired sites of mutagenesis. Fusion PCR was then performed for each mutation by adding both the 5’ and 3’ amplified fragments into the same reaction, along with the forward primers for the 5’ sequences and reverse primers for the 3’ sequences, so as to generate full length sequence containing the desired point mutations. These sequences were then inserted into plasmid pUCGH at the BamHI restriction site, followed by insertion of the same *pkaC1* downstream flanking sequence used for PkaC1-GFP labeling described above, again into the SbfI-HindIII restriction site, downstream of the hygromycin B resistance cassette. Plasmids were linearized with HindIII and transformed into the *akuB^KU80^* strain, followed by hygromycin B resistance screening as above. Transformants were verified for homologous integration by PCR and fluorescence microscopy. Recombinant strains were sequenced to confirm *pkaC1* mutation. For S175 mutations, the forward primer used for 5’ sequence amplification for fusion PCR was PkaC1-ABhelixmut-F(BamHI). For T331, T333 and T337 mutations, the 5’ sequence forward primer was PkaC1-aloopmut-F(BamHI). For all mutations, the reverse primer for 3’ sequence was PkaC1-GFP-R(BamHI), so that mutated PkaC1 isoforms also possessed 3’ GFP labels. For S175 to alanine mutation (S175A), the reverse primer for 5’ fusion sequence amplification was PkaC1-S175A-R, while the overlapping forward primer for 3’ fusion sequence amplification was PkaC1-S175A-F. The corresponding fusion primers for glutamate substitution at this site (S175E) were PkaC1-S175E-F and PkaC1-S175E-R. Respective corresponding fusion primers for mutations of T331, T333 and T337 to alanine (A) and glutamate (E) were as follows: PkaC1-T331A-F and PkaC1-T331A-R for T331A, PkaC1-T331E-F and PkaC1-T331E-R for T331E, PkaC1-T333A-F and PkaC1-T333A-R for T333A, PkaC1-T333E-F and PkaC1-T333E-R for T333E, PkaC1-T337A-F and PkaC1-T337A-R for T337A, PkaC1-T337E-F and PkaC1-T337E-R for T337E.

N-terminal RFP-labeling of PkaR was accomplished by insertion of the PkaR coding region amplified from AF293 genomic DNA (primers PkaR-RFP-F(BamHI) and PkaR-RFP-R(BamHI)) 3’ to - and in frame with - the *rfp* coding region (lacking a stop codon) of the pJW24-RFP vector at the BamHI site, followed by the insertion of upstream (primers PkaR-up-F(SacI) and PkaR-up-R(NotI)) and downstream (primers PkaR-down-F(EcoRI) and PkaR-down-R(SalI)) flanking sequences of about 1.2 kb each, into SacI-NotI and EcoRI-SalI restriction sites, respectively. Linearized construct was transformed into *akuB^KU80^* *pyrG*- auxotrophic strain and transformants selected on SMM agar were screened for homologous recombination via PCR and DNA sequencing.

Ectopic expression of native PkaC1 and PkaC1 containing the T333A point mutation was accomplished by amplification of the *pkaC1* coding region genomic DNA from strains AF293 and PkaC1-T333A-GFP, respectively (Table S2), using primers PkaC1-otef-F(BamHI) and PkaC1-GFP-R(BamHI), followed by insertion of amplified fragments into the BamHI site of plasmid pUCGH, immediately 3’ to the *otef* promoter region (3). Circular plasmid constructs were then used to transform strain *akuB^KU80^*, followed by hygromycin B selection and screening via fluorescence microscopy, PCR and sequencing to confirm transformations as above.

Combined expression of RFP-labeled PkaR with either GFP-labeled native PkaC1 or T333A-mutated PkaC1 was accomplished by transformation of strain RFP-PkaR (Table S2) with circular constructs as described above for ectopic expression of the two GFP-labeled isoforms of PkaC1. Screening of transformants was performed as described above. All strains generated and used in this study are listed in Table S1.

**REFERENCES**

1. **Steinbach WJ, Cramer RAJ, Perfect BZ, Asfaw YG, Sauer TC, Najvar LK, Kirkpatrick WR, Patterson TF, Benjamin DKJ, Heitman J, Perfect JR.** 2006. Calcineurin controls growth, morphology, and pathogenicity in Aspergillus fumigatus. Eukaryot Cell **5 SRC - GoogleScholar:**1091-1103.

2. **Juvvadi PR, Belina D, Soderblom EJ, Moseley MA, Steinbach WJ.** 2013. Filamentous fungal-specific septin AspE is phosphorylated in vivo and interacts with actin, tubulin and other septins in the human pathogen Aspergillus fumigatus. Biochem Biophys Res Comm **431 SRC - GoogleScholar:**547-553.

3. **Spellig T, Bottin A, Kahmann R.** 1996. Green fluorescent protein (GFP) as a new vital marker in the phytopathogenic fungus Ustilago maydis. Mol Gen Genet **252:**503-509.
