# Supplementary material for: A Novel Phosphoregulatory Switch Controls the Activity and Function of the Major Catalytic Subunit of Protein Kinase A in Aspergillus fumigatus
Source: mBio. 2017 Feb 7;8(1):e02319-16. doi: 10.1128/mBio.02319-16 (PMC5296607; doi:10.1128/mBio.02319-16)
Supplement: TABLE S2 [file mbo001173178st2.docx]

**Table S2. Primers used in this study**

| Primer name | Primer sequence (5’ to 3’) |
| --- | --- |
| PkaC1-upstream-F(SalI) | CATGCTACGTCGACTCACCCAAACTC |
| PkaC1-upstream-R(EcoRI) | ATGGACCATAGAATTCGTGAGTGGCAG |
| PkaC1-downstream-F(NotI) | TGACTGGAGGCGGCCGCGATACAAATG |
| PkaC1-downstream-R(SacI) | AGAGTATATGAGAGCTCCATGCTCCC |
| PkaC1-GFP-F(BamHI) | TTTAACTCTCGGATCCTCGATGCC |
| PkaC1-GFP-R(BamHI) | ATGTCGTATATTGGATCCGAAGTCTGGGAAAAGA |
| PkaC1-downstream-F(SbfI) | ACGATACACCTGCAGGAGCCTCCACTCTAGG |
| PkaC1-downstream-R | GAGAGGAAGGCGTACGGTGG |
| PkaC1-ABhelixmut-F(BamHI) | TGAAGTCACCGGATCCGAGGCTTCGTAGGG |
| PkaC1-S175A-R | GGTGAAATCGTCTAGGGCATATTT-CCCTTTCGTAGTGCG |
| PkaC1-S175A-F | CGCACTACGAAAGGGAAATATGCCC-TAGACGATTTCACC |
| PkaC1-aloopmut-F(BamHI) | TAAGATATGGATCCCAAGAGGACATTGGGC |
| PkaC1-T333A-F | CCTGATATCACATGGGCTCTTTGCGGAACG |
| PkaC1-T333A-R | CGTTCCGCAAAGAGCCCATGTGATATCAGG |
| PkaC1-T331A-F | CCTGATATCGCATGGACTCTTTGCGGAACG |
| PkaC1-T331A-R | CGTTCCGCAAAGAGTCCATGCGATATCAGG |
| PkaC1-T337A-F | GGACTCTTTGCGGAGCGCCCGATTATCTTGC |
| PkaC1-T337A-R | GCAAGATAATCGGGCGCTCCGCAAAGAGTCC |
| PkaC1-T331E-F | CCTGATATCGAATGGACTCTTT-GCGGAACGCCCGATTATCTTGCTCC |
| PkaC1-T331E-R | GGAGCAAGATAATCGGGCGTT-CCGCAAAGAGTCCATTCGATATCAGG |
| PkaC1-T333E-F | CCTGATATCACATGGGAACTTT-GCGGAACGCCCGATTATCTTGCTCC |
| PkaC1-T333E-R | GGAGCAAGATAATCGGGCGTT-CCGCAAAGTTCCCATGTGATATCAGG |
| PkaC1-T337E-F | CCTGATATCACATGGACTCTTT-GCGGAGAGCCCGATTATCTTGCTCC |
| PkaC1-T337E-R | GGAGCAAGATAATCGGGCTCT-CCGCAAAGAGTCCATGTGATATCAGG |
| PkaC1-S175E-F | CGCACTACGAAAGGGAAATATGAGCTAGACGATTTCACC |
| PkaC1-S175E-R | GGTGAAATCGTCTAGCTCATA-TTTCCCTTTCGTAGTGCG |
| PkaC1-ups-F2(KpnI) | GTAATGAAGGTACCAAGCTAGAGGCTTCG |
| PkaC1-upstream-R(EcoRI) | ATGGACCATAGAATTCGTGAGTGGCAG |
| PkaC1-otef-F(BamHI) | TTTAACTCTCGGATCCTCGATGCCGACTTTAGG |
| PkaC1-upstream-R(EcoRI) | ATGGACCATAGAATTCGTGAGTGGCAG |
| PkaR-up-F(SacI) | TTTCTTGAGCTCGGCGTTGATTGTTCCC |
| PkaR-up-R(NotI) | TTGGCTGTGCGGCCGCAGATGGTACAG |
| PkaR-RFP-F(BamHI) | TTACAGCCGGATCCATGGCTGATAGCTC |
| PkaR-RFP-R(BamHI) | AGTCTATCGGGATCCTCACGAGGGCG |
| PkaR-down-F(EcoRI) | TAAAGTGGTTGAATTCGAGACATGCTTGG |
| PkaR-down-R(SalI) | AGCGGTGCTGTCGACGAGGACCAGG |
| PkaC1-RT-F | TCGGTTGATTGGTGGTCACTTGG |
| PkaC1-RT-R | TAGGGCGGGTACTTGACACGG |
| 5’ β-Tubulin | TTCCCAACAACATCCAGACC |
| 3’ β-Tubulin | CGACGGAACATAGCAGTGAA |
